# Renogrit selectively protects against cisplatin-induced injury in human renal tubular cells and in *Caenorhabditis elegans* by harmonizing apoptosis and mitophagy

**DOI:** 10.1038/s41598-024-69797-3

**Published:** 2024-08-21

**Authors:** Acharya Balkrishna, Vivek Gohel, Nishit Pathak, Monali Joshi, Rani Singh, Ankita Kumari, Rishabh Dev, Anurag Varshney

**Affiliations:** 1 Drug Discovery and Development Division, Patanjali Research Foundation, NH-58, Haridwar, Uttarakhand 249405 India; 2 Department of Allied and Applied Sciences, University of Patanjali, Patanjali Yog Peeth, Roorkee-Haridwar Road, Haridwar, Uttarakhand 249405 India; 3 Patanjali Yog Peeth (UK) Trust, 40 Lambhill Street, Kinning Park, Glasgow, G411AU UK; 4 Special Centre for Systems Medicine, Jawaharlal Nehru University, New Delhi, 110067 India

**Keywords:** Kidney diseases, Autophagy

## Abstract

Cisplatin-induced nephrotoxicity restricts its clinical use against solid tumors. The present study elucidated the pharmacological effects of Renogrit, a plant-derived prescription medicine, using cisplatin-induced human renal proximal tubular (HK-2) cells and *Caenorhabditis elegans*. Quantification of phytochemicals in Renogrit was performed on HPTLC and UHPLC platforms. Renogrit was assessed in vitro in HK-2 cells post-exposure to clinically relevant concentration of cisplatin. It was observed that renoprotective properties of Renogrit against cisplatin-induced injury stem from its ability to regulate renal injury markers (KIM-1, NAG levels; NGAL mRNA expression), redox imbalance (ROS generation; GST levels), and mitochondrial dysfunction (mitochondrial membrane potential; SKN-1, HSP-60 expression). Renogrit was also found to modulate apoptosis (EGL-1 mRNA expression; protein levels of p-ERK, p-JNK, p-p38, c-PARP1), necroptosis (intracellular calcium accumulation; RIPK1, RIPK3, MLKL mRNA expression), mitophagy (lysosome population; mRNA expression of PINK1, PDR1; protein levels of p-PINK1, LC3B), and inflammation (IL-1β activity; protein levels of LXR-α). More importantly, Renogrit treatment did not hamper normal anti-proliferative effects of cisplatin as observed from cytotoxicity analysis on MCF-7, A549, SiHa, and T24 human cancer cells. Taken together, Renogrit could be a potential clinical candidate to mitigate cisplatin-induced nephrotoxicity without compromising the anti-neoplastic properties of cisplatin.

## Introduction

Cancer is one of the leading causes of death. It is not only responsible for mortality but is also associated with morbidities arising from the disease itself and related adverse effects of chemotherapy^1,2^. Nephrotoxicity is a major adverse effect among cancer patients treated with conventional cytotoxic agents or targeted therapies which often leads to lengthy hospitalizations^3^. Cisplatin (cis-diamminedichloroplatinum II) is an indispensable chemotherapeutic agent commonly used for the treatment of various malignant diseases like, esophageal, testicular, head and neck, bladder, ovarian, breast, cervical, uterine, stomach, small-cell and non-small cell lung cancers^4^. However, the most common adverse effect observed in patients treated with cisplatin is nephrotoxicity which occurs due to its accumulation and biotransformation to highly reactive thiols in kidneys^5,6^. Clinically, cisplatin-induced nephrotoxicity is observed in nearly one-third of patients undergoing treatment. Approximately, 40% of these patients fail to complete the therapy due to renal impairment thereby affecting the patients’ survival rate^7^. The pathophysiology of cisplatin-induced nephrotoxicity involves oxidative stress, mitochondrial dysfunction, apoptosis, inhibition of mitophagy, and inflammation^8,9^. At present, cisplatin-induced nephrotoxicity is mitigated mainly by mannitol-induced forced diuresis but this intervention often leads to over-diuresis and dehydration. Hence, there is a large unmet medical need for renoprotective agents that could be used as adjuvant therapy for cisplatin-treated patients^10^. The proximal tubule cell line, human kidney 2 (HK-2) cells, has been widely accepted as a cellular tool for in vitro toxicological studies, including cisplatin-induced nephrotoxicity. These HK-2 cells show morphological and functional similarities to the primary human proximal tubule epithelial cells^11–14^. In the other spectrum of biological complexities, *Caenorhabditis elegans* has been a well-explored whole-organism model to evaluate cisplatin-induced toxicity and associated mitochondrial dysfunction^15–18^.

Renoprotective agents from ethnopharmacological origins may serve as an attractive option to mitigate cisplatin-induced nephrotoxicity due to their known efficacies, better safety margins, advantageous pleiotropisms, and cost-effectiveness^8^. The plant-derived medicine, Renogrit is routinely prescribed to treat kidney disorders, in India. Renogrit, an Ayurvedic herbal medicine, composed of extracts from seven herbs namely, *Achyranthes aspera* L. (Apamarg), *Saxifraga ligulata* Murray (Pashanbhed), *Butea frondosa Roxb.* ex Willd. (Palash), *Crateva nurvala* Buch.-Ham. (Varun), *Boerhavia diffusa* L. (Punarnavamool), *Cichorium intybus* L. (Kasni), and *Tribulus terrestris* L. (Gokharu). These herbs are known to possess robust anti-oxidant^19,20^, anti-apoptotic^21^, and anti-inflammatory^22,23^ properties. The present study investigated the pharmacological effects of Renogrit in cisplatin-induced HK-2 cells and various strains of *C*. *elegans*. An extensive phytochemical profiling of Renogrit was carried out by HPTLC and UHPLC analysis and subsequently, its biological activities were evaluated. In the in vitro studies, biomarkers of cisplatin-induced nephrotoxicity like tubular cell cytotoxicity, NAG and KIM-1 levels, NGAL mRNA expression, oxidative stress, loss of mitochondrial membrane potentials, apoptosis, necroptosis, defective mitophagy, and inflammation were evaluated post Renogrit treatment. In addition, the ability of Renogrit to prevent nephrotoxicity without affecting the anti-cancer properties of cisplatin was evaluated. Furthermore, the effects of Renogrit were studied in vivo, on cisplatin-induced N2, SYS81, CL2166, and SJ4058 strains of *C*. *elegans*. In the parallel experiments, N-acetylcysteine (NAC), known to alleviate cisplatin-induced nephrotoxicity in preclinical studies,was used as an experimental control^3^.

## Results

### Phytochemical profile of Renogrit

**Figure 1:**
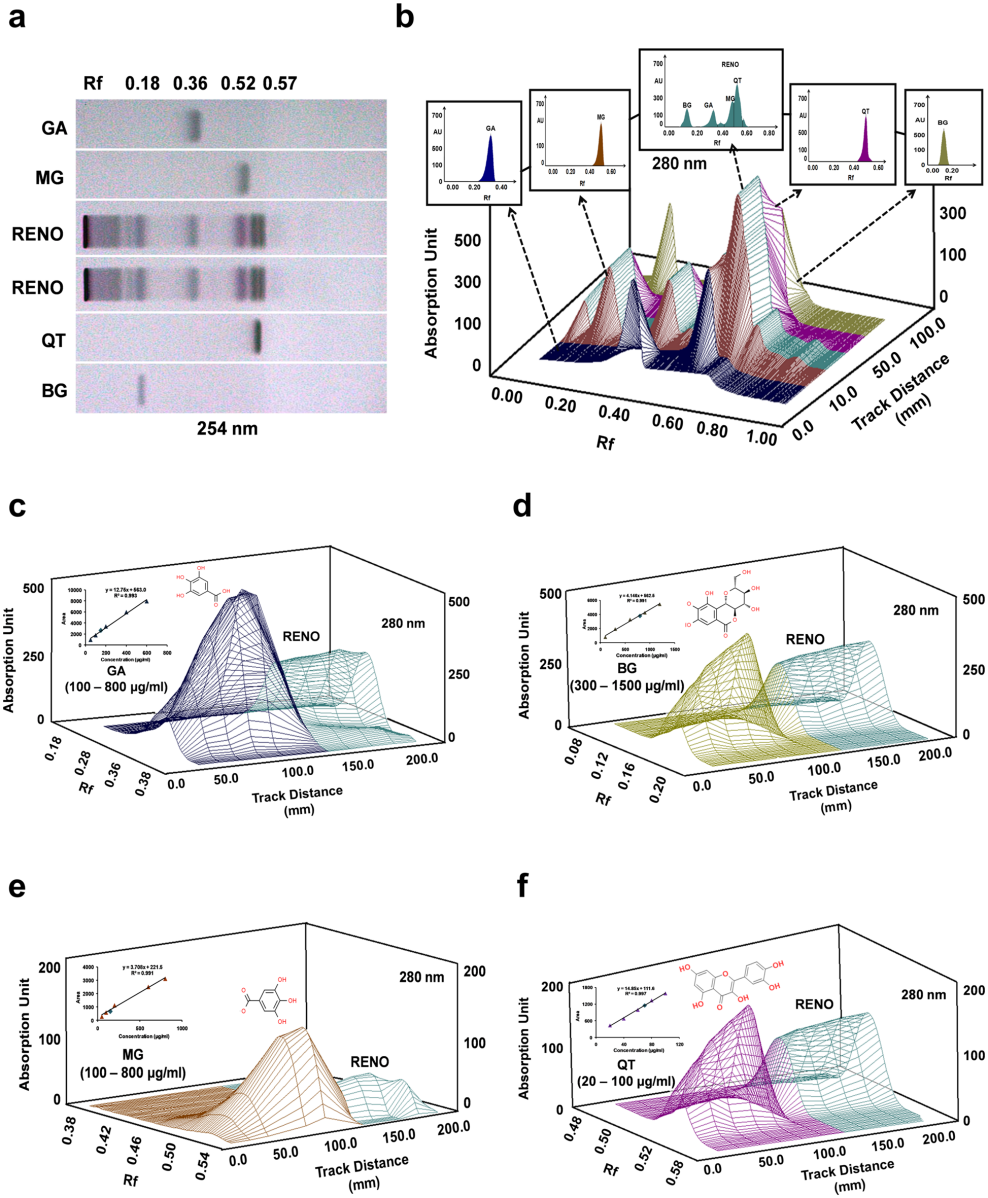
HPTLC profile of phytochemicals present in Renogrit. (**a**) Developed HPTLC plate under exposure to UV light (λ = 254 nm) with Retardation factor (Rf) of Gallic acid (GA), Methyl gallate (MG), Renogrit (RENO), Quercetin (QT), and Bergenin (BG). (**b**) 3D- HPTLC chromatogram of separated peaks showing presence of Renogrit’s marker compounds, GA (Rf = 0.36), MG (Rf = 0.52), QT (Rf = 0.57), and BG (Rf = 0.18) acquired at 280 nm using toluene: ethyl acetate: methanol: formic acid (5:4:1:0.2, by volume) as a developing system using densitometric scan. Quantitative analysis of the marker compounds in Renogrit was done based on the linearity plot and peak tables of (**c**) GA, (**d**) BG, (**e**) MG, and (**f**) QT along with RENO attained at 280 nm. Quantification of the phytochemicals has been mentioned in Table 1.

**Figure 2:**
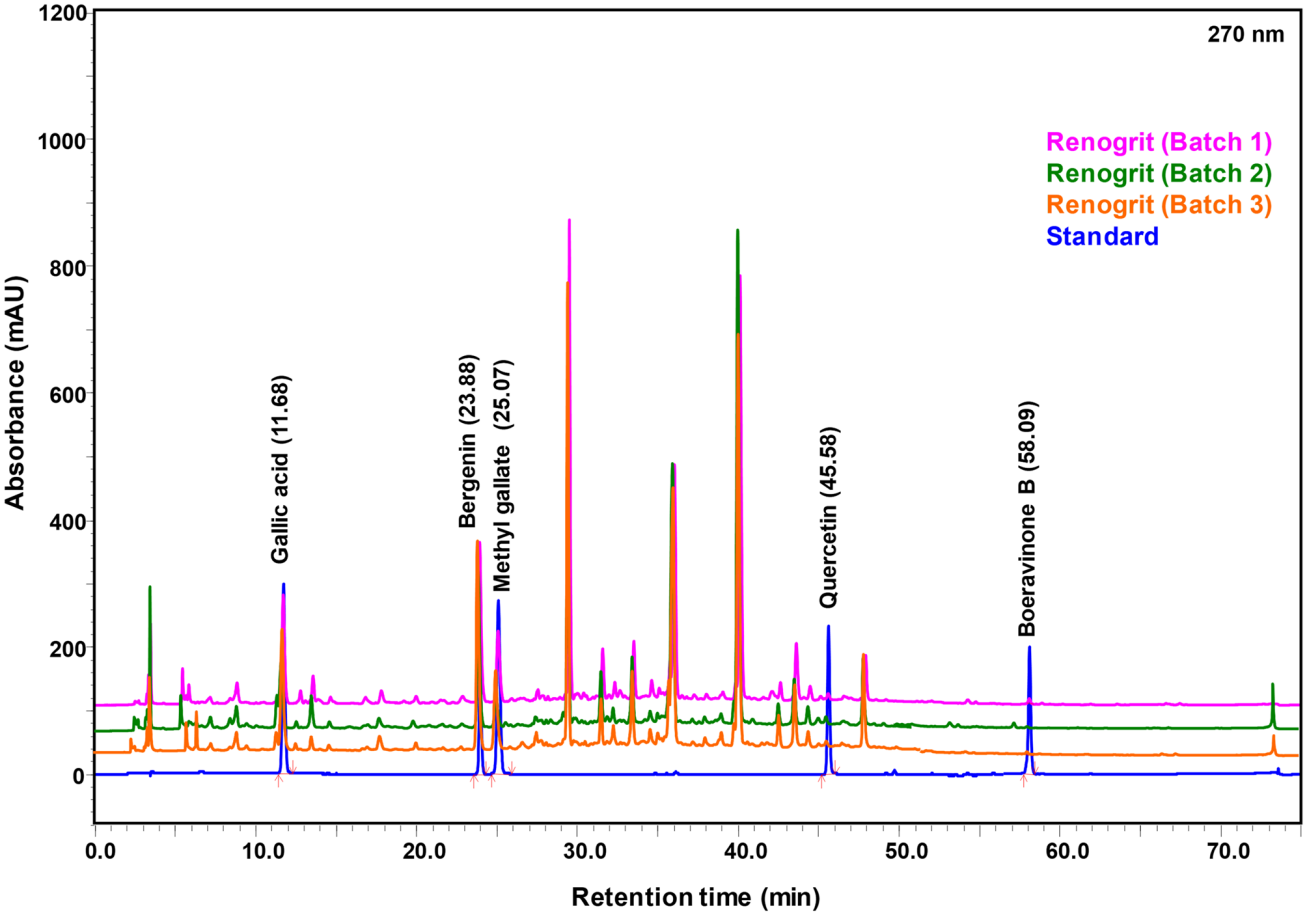
UHPLC based analysis of phytoconstituents present in Renogrit. Phytoconstituents of 3 random batches of Renogrit (Batch 1- CHIH/RENA/0222/2304; Batch 2- CHIH/RENA/0222/2321; Batch 3- CHIH/RENA/0322/2431) analysed for comparative quantification of the marker phytochemical compounds. Overlap chromatogram of standard mix (Blue line), and Batch 1 (Pink line), Batch 2 (Green line), Batch 3 (Orange line) of Renogrit. The phytoconstituents namely Gallic acid, Bergenin, Methyl gallate, Quercetin, and Boeravinone B were quantified by their respective reference standards at 270 nm. The quantification of the evaluated phytoconstituents is mentioned in Table 1.

Primary screening of phytochemicals in Renogrit was performed by HPTLC method (Fig. 1). The co-chromatography of Renogrit (RENO) along with reference standards showed the presence of polyphenols namely gallic acid (GA), Methyl gallate (MG), Quercetin (QT), and Bergenin (BG). The retardation factor (Rf) obtained of the phytochemicals were- GA (0.36), MG (0.52), QT (0.57), and BG (0.18) (Fig. 1a,b). Furthermore, quantification of the observed phytochemicals in Renogrit was done on the basis of linear plots of reference standards as displayed in the peak tables (Fig. 1c–f). HPTLC-based quantitative analysis of the identified phytometabolites in Renogrit is provided in Table 1. UHPLC-based analysis of three different batches of Renogrit along with reference standards displayed the presence of Gallic acid, Bergenin, Methyl gallate, Quercetin, and Boeravinone B (Fig. 2). Quantification of the phytometabolites from all the three batches of Renogrit is mentioned in Table 1. A similarity was observed in the values of phytochemicals quantified by HPTLC and UHPLC. However, as HPTLC has a limitation of sensitivity, the detection and quantification of Boeravinone B was viable by UHPLC analysis only (Fig. 2).

**Table 1:** Phytochemicals present in Renogrit as deciphered from chromatograms shown in Figs. 1 and 2.

S. no	Compound	Structure of compound	Quantity in Renogrit (µg/mg) as per HPTLC	Quantity in Renogrit (n = 3) (mean ± S.E.M; µg/mg) as per UHPLC
1	Gallic acid		2.38	2.47 ± 0.52
2	Bergenin		8.42	8.88 ± 0.65
3	Methyl gallate		2.22	1.86 ± 0.43
4	Quercetin		0.17	0.17 ± 0.04
5	Boeravinone B		–	0.05 ± 0.01

### Renogrit treatment decreased cell injury in cisplatin-induced HK-2 cells

**Figure 3:**
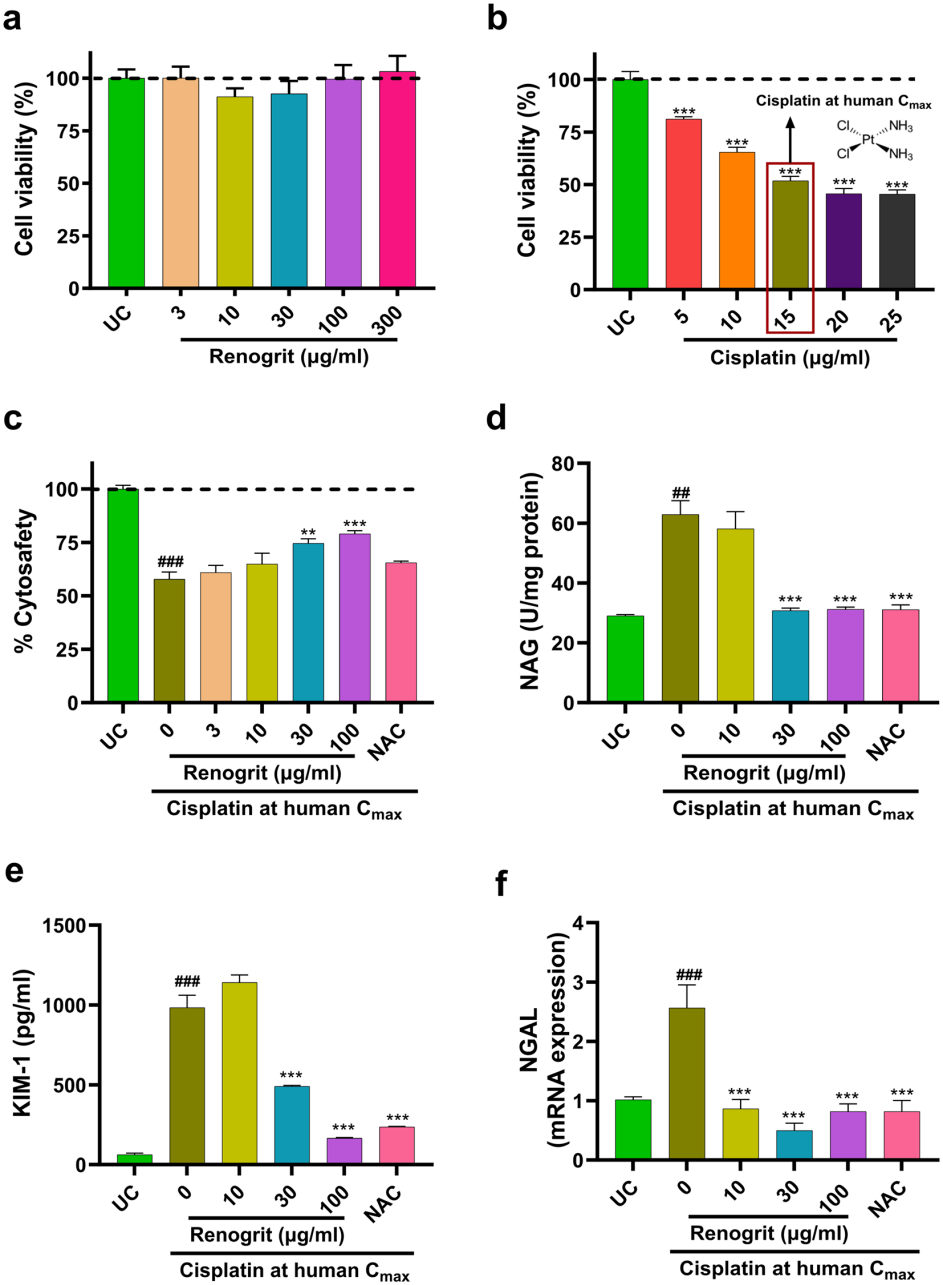
Renogrit decreased cisplatin-induced injury in renal proximal tubular cells (HK-2). (**a**) Renogrit was found to be safe upto concentration of 300 µg/ml in HK-2 cells. (**b**) Cisplatin (5–25 µg/ml) induction in HK-2 cells decreased the cell viability. The cisplatin concentration of 15 µg/ml was used for further experiments as it is the maximum serum concentration (C_max_) of cisplatin found in human subjects. (**c**) Renogrit (3–100 µg/ml) enhanced the viability of HK-2 cells induced with cisplatin at human C_max_ (15 µg/ml). N-acetyl cysteine (NAC) at concentration of 2 mM was used as the positive control. Renogrit treatment reduced the cisplatin stimulated release of nephrotoxicity biomarkers, (**d**) NAG, (**e**) KIM-1, and (**f**) mRNA expression of NGAL. Data represented as mean ± S.E.M. ^###^ and ***p < 0.001; ^##^ and **p < 0.01.

Treatment of HK-2 cells with Renogrit (3–300 µg/ml) for 24 h showed no significant change in cell viability (Fig. 3a) compared to untreated control (UC). Cisplatin (5–25 µg/ml) induced HK-2 cells showed a significant (p < 0.001) concentration-dependent decline in cell viability compared to UC (Fig. 3b). Cisplatin at 15 µg/ml was used for further analysis as it is a clinically relevant dose of cisplatin (C_max_ of cisplatin as detected from the blood plasma of patients)^24^. Renogrit (3–100 µg/ml) treatment led to a concentration-dependent rise in the viability of cisplatin-induced HK-2 cells which was significant in cells treated with 30 µg/ml (p < 0.01) and 100 µg/ml (p < 0.001) of Renogrit. NAC (2 mM) treated group showed a marginal improvement in cell viability (Fig. 3c). Later, the biomarkers of cisplatin-induced kidney injury namely NAG, KIM-1, and NGAL were evaluated^25^. It was observed that cisplatin-induced HK-2 cells showed a significant (p < 0.01) increase in NAG levels. Cisplatin-induced cells in the presence of Renogrit (30 and 100 µg/ml) showed normalized levels of NAG release (Fig. 3d). The released KIM-1 levels were also significantly (p < 0.001) increased in cells induced with cisplatin compared to control. Cisplatin-induced HK-2 cells in presence of Renogrit (30 and 100 µg/ml) treatment showed a significant (p < 0.001) decline in released KIM-1 levels (Fig. 3e). Furthermore, Renogrit (10, 30, and 100 µg/ml) treated cells significantly (p < 0.001) decreased the overexpression of NGAL gene (Fig. 3f). NAC treatment of cisplatin-induced cells also led to the decline of NAG, KIM-1 release and NGAL gene upregulation (Fig. 3d–f).

### Renogrit treatment reduced markers of oxidative stress in cisplatin-induced HK-2 cells

**Figure 4:**
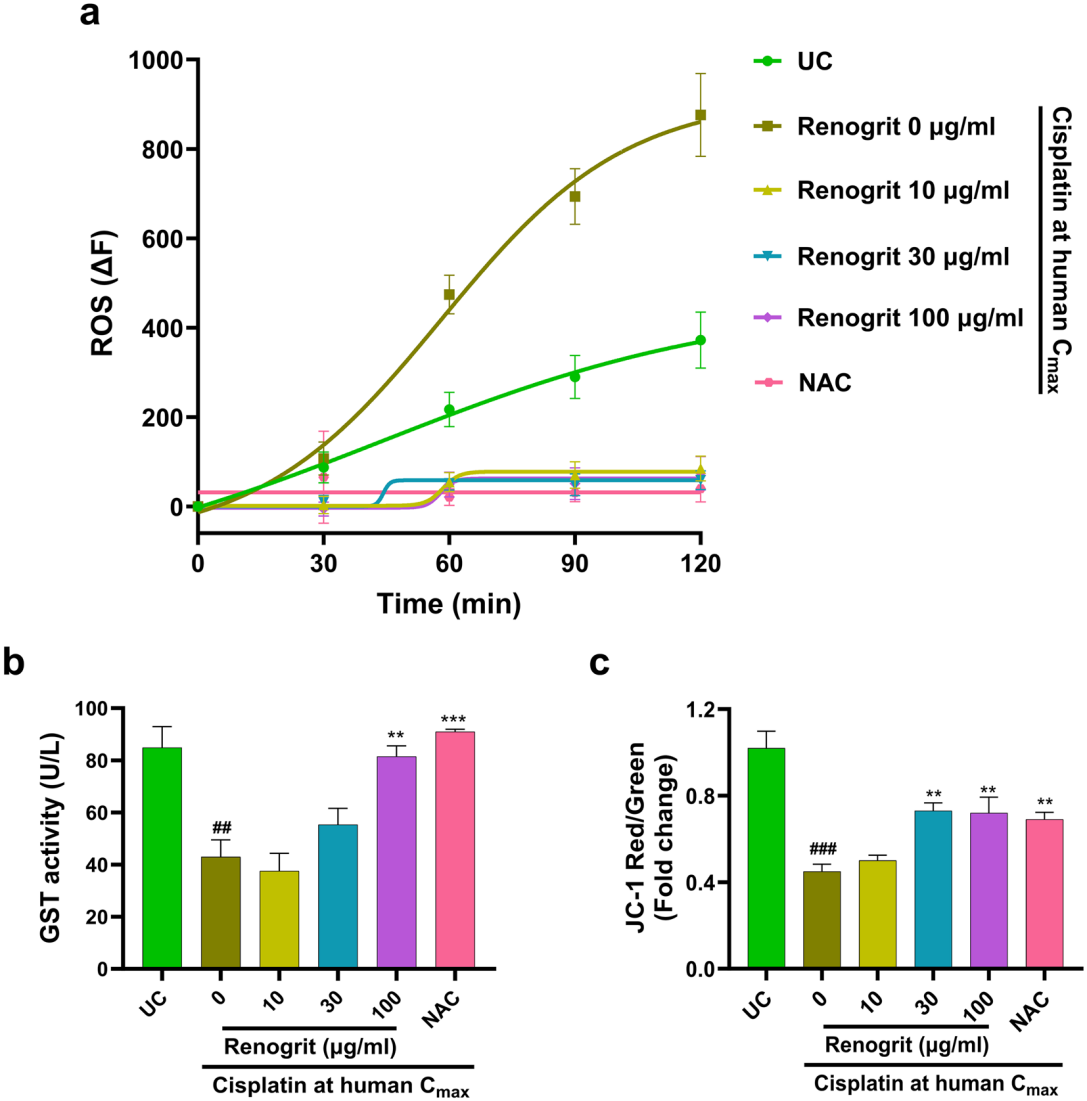
Renogrit decreased cisplatin-induced oxidative stress and mitochondrial dysfunction in HK-2 cells. (**a**) The reactive oxygen species (ROS) burst generated in response to HK-2 cells induced with cisplatin at human C_max_ (15 µg/ml) was inhibited in presence of Renogrit (10–100 µg/ml). Prior to fluorescence measurements, H_2_DCFDA stained HK-2 cells were simultaneously treated with cisplatin and Renogrit or NAC (2 mM) and the converted DCF was evaluated for 120 min. (**b**) Renogrit treatment normalized the GST levels in HK-2 cells that were altered in response to cisplatin induction. (**c**) Renogrit treatment enhanced the mitochondrial membrane potential of HK-2 cells which was decreased in response to cisplatin induction as observed by ratiometric fluorescence measurements of cells stained with JC-1. Data represented as mean ± S.E.M. ^###^ and ***p < 0.001; ^##^ and **p < 0.01.

Real-time analysis of ROS generation up to 120 min showed that HK-2 cells treated with Renogrit (10, 30, and 100 µg/ml) displayed no production of ROS in response to cisplatin induction. However, cisplatin-induced cells showed a steady rise in the generation of ROS (Fig. 4a). The anti-oxidant activity of GST in kidney tissues is known to be suppressed by cisplatin^26^. Here, the significant (p < 0.01) decrease in the activity of GST enzyme in cisplatin-induced HK-2 cells got normalized in a concentration-dependent manner in the presence of Renogrit (Fig. 4b). An increase in ROS generation alters the mitochondrial membrane potential^27^. It was observed that cisplatin-induced cells showed a significant (p < 0.001) decrease in mitochondrial membrane potential as evaluated by ratiometric analysis of JC-1 dye aggregates. Renogrit-treated cells showed a significant (p < 0.01) increase in mitochondrial membrane potential compared to only cisplatin-induced cells (Fig. 4c). NAC (2 mM) treated cisplatin-stimulated cells also decreased the markers of oxidative stress (Fig. 4a–c).

### Renogrit treatment lowered apoptosis in cisplatin-induced HK-2 cells

**Figure 5:**
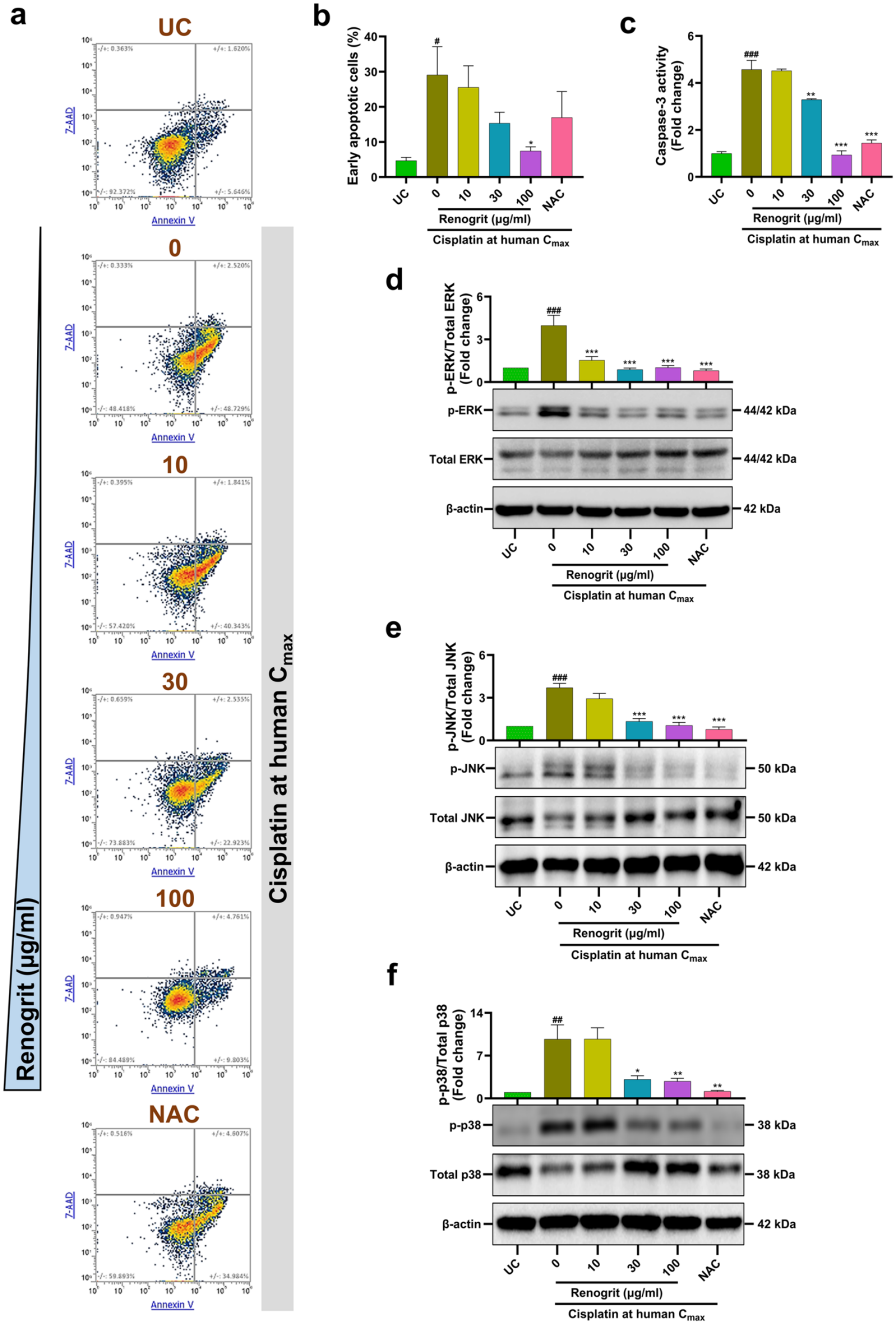
Renogrit decreased cisplatin-induced apoptotic cell-death by modulation of MAPKs in HK-2 cells. (**a**) Flow cytometry density plots of Annexin V-7AAD stained HK-2 cells induced with cisplatin at human C_max_ (15 µg/ml) and treated with Renogrit (10–100 µg/ml) or NAC (2 mM). Percentage of cells with Annexin V and/or 7AAD are mentioned in the plots. (**b**) Percentage of early apoptotic cells (positive for Annexin V stain) in untreated control (UC) and cisplatin at human C_max_ (15 µg/ml) with and without Renogrit (10–100 µg/ml) or NAC (2 mM) treatment. (**c**) Relative caspase-3 activity in treated and untreated HK-2 cells. (**d**–**f**) Representative western blots of phosphorylated and total MAPK family proteins and relative quantification of phosphorylated ERK, JNK, and p-38 respectively. Western blot analysis of proteins in the mitogen-activated protein kinase (MAPK) pathway of treated and untreated HK-2 cells showed that Renogrit (10–100 µg/ml) decreased the phosphorylation of (**d**) ERK, (**e**) JNK, and (**f**) p-38. Data represented as mean ± S.E.M. ^###^ and ***, p < 0.001; ^##^ and **p < 0.01; # and *p < 0.05.

Apoptosis is one of the pathological features of cisplatin-induced nephrotoxicity^28^. Flow cytometry based analysis of apoptosis by Annexin V-7AAD staining revealed that Renogrit (100 µg/ml) treatment led to a significant (p < 0.05) decrease in the population of cells in early apoptosis which were significantly (p < 0.05) increased in cisplatin-induced cells (Fig. 5a,b). This observation was further cemented by the significant (p < 0.001) inhibition of caspase-3 activity in Renogrit treated cells induced with cisplatin (Fig. 5c). Cisplatin-stimulated renal tubular damage involves activation of proteins involved in MAPK signaling^29^. Phosphorylation of the three major MAPKs which regulate the apoptosis pathway namely ERK, JNK, and p38 significantly (p < 0.01) increased upon cisplatin-stimulation of HK-2 cells. Renogrit treatment in these cells led to a significant (p < 0.01) decline in phosphorylation hitherto activation of the MAPKs (Fig. 5d–f). NAC (2 mM) treatment of the cisplatin-stimulated HK-2 cells also modulated the markers of apoptosis (Fig. 5a–f).

### Renogrit treatment reduced necroptosis in cisplatin-induced HK-2 cells

**Figure 6:**
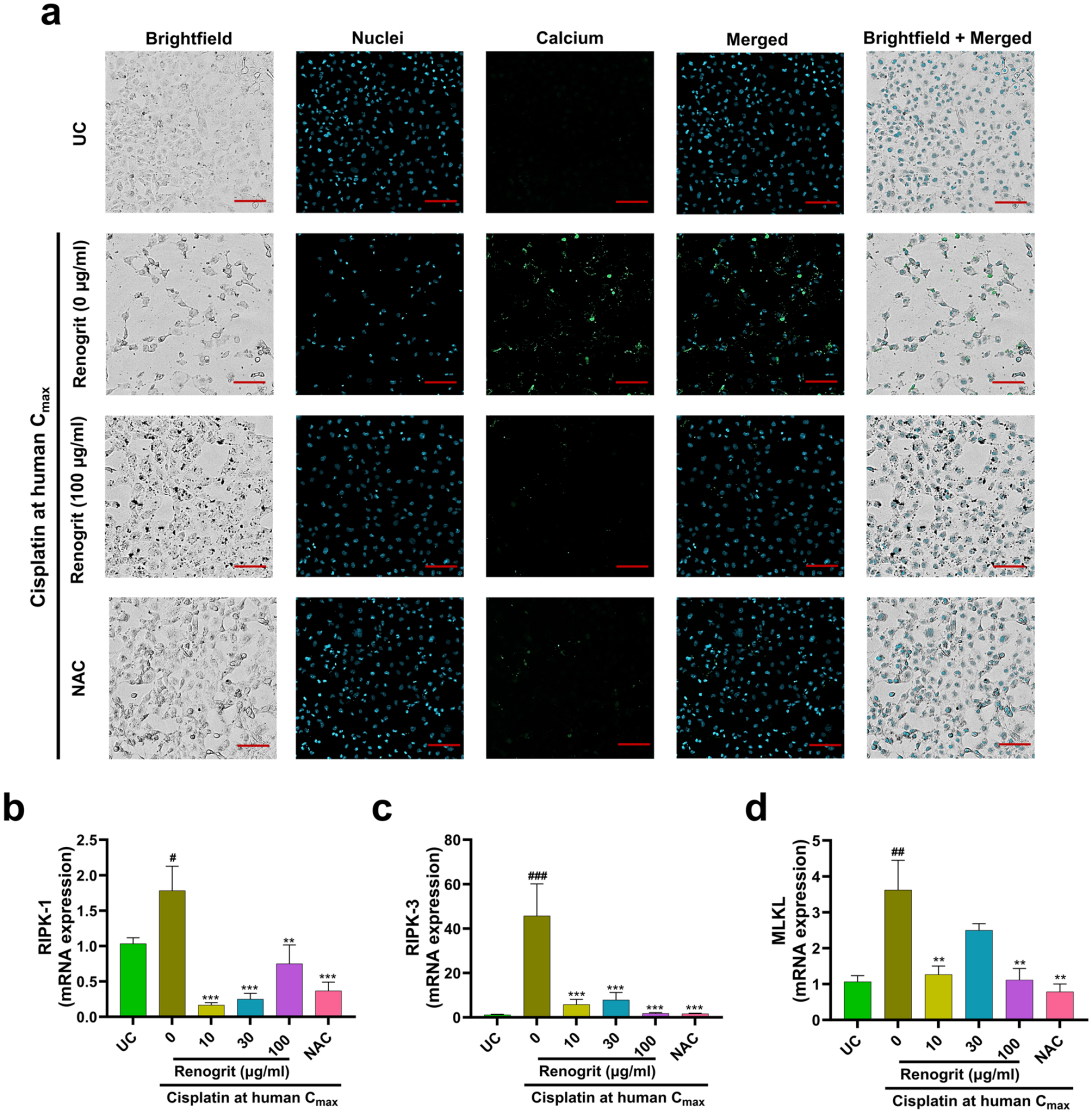
Renogrit decreased cisplatin-induced cytosolic calcium accumulation and RIPK1-RIPK3-MLKL overexpression in HK-2 cells. (**a**) Image of Hoechst 33342 (nuclei, blue) and Fluo-4 AM (calcium, green) stained HK-2 cells induced with cisplatin at human C_max_ (15 µg/ml) and treated with Renogrit (100 µg/ml) or NAC (2 mM). Magnification = 100 × . Scale bar (red) = 40 µm. Relative mRNA expression of genes involved in necroptosis namely (**b**) RIPK1, (**c**) RIPK2, and (**d**) MLKL of treated and untreated HK-2 cells. ### and ***, p < 0.001; ## and **p < 0.01; #p < 0.05.

Calcium overload in the cytoplasm is a major biomarker of cisplatin-induced necroptosis which occurs in line with RIPK-1, RIPK-3, and MLKL (core components of necroptotic pathway) complex formation^7,30^. Calcium accumulation was observed in cisplatin-induced HK-2 cells which declined in the presence of Renogrit (100 µg/ml) or NAC (Fig. 6a). The significant (p < 0.05) upregulation in the gene expression of RIPK-1, RIPK-3, and MLKL in response to cisplatin induction in HK-2 cells was normalized upon Renogrit (100 µg/ml) or NAC treatment (Fig. 6b–d).

### Renogrit treatment enhanced lysosome population and mitophagy in cisplatin-induced HK-2 cells

**Figure 7:**
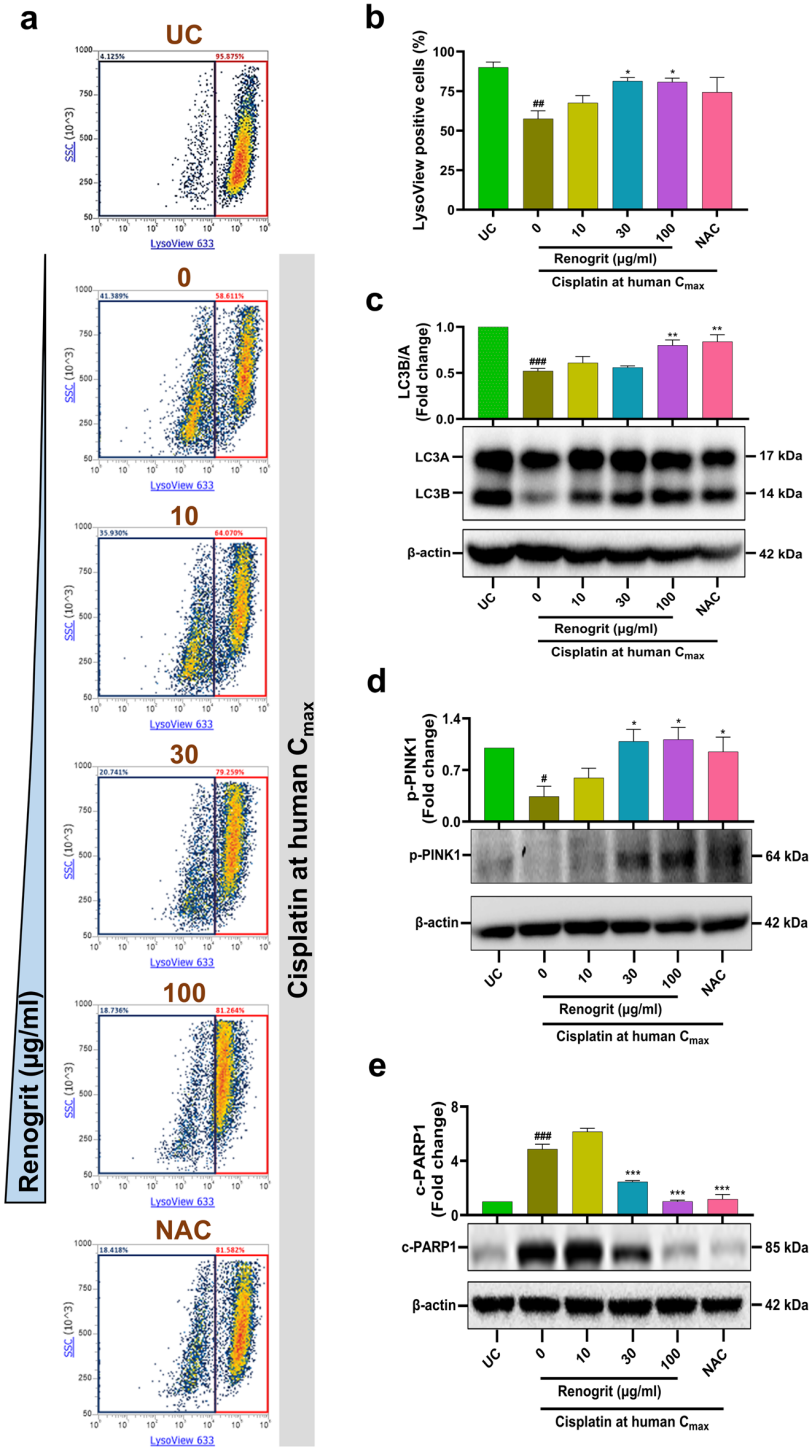
Renogrit prevented cisplatin-induced cell-death by modulation of mitophagy and phosphorylation of PARP-1 protein in HK-2 cells. (**a**) Flow cytometry density plots of LysoView 633 stained HK-2 cells induced with cisplatin at human C_max_ (15 µg/ml) and treated with Renogrit (10–100 µg/ml) or NAC (2 mM). Percentage LysoView 633 (− ve, blue; + ve, red) cells are mentioned in the density plots. (**b**) LysoView 633 positive cells (%) in treated and untreated HK-2 cells. (**c**–**e**) Representative western blots and relative quantification of proteins involved in the mitophagy and cell-death pathway showed that Renogrit (10–100 µg/ml) increased (**c**) LC3B/A conversion, (**d**) phosphorylation of PINK1, and decreased (**e**) cleaved PARP-1. Data represented as mean ± S.E.M. ^###^ and ***p < 0.001; ^##^ and **p < 0.01; ^#^ and *p < 0.05.

In renal tubular cells, cisplatin-induction is known to decrease lysosome numbers which leads to defects in mitophagy and eventually renal cell apoptosis^9,31^. In the current study also, cisplatin-induced HK-2 cells revealed a significant (p < 0.01) decline in the lysosome population as observed from flow cytometry based analysis. When these cells were treated with Renogrit a concentration-dependent recovery of lysosome number was observed which was significant (p < 0.05) at 30 and 100 µg/ml concentration. NAC treatment also showed a noticeable rise in the lysosome population (Fig. 7a,b). The conversion of LC3B from LC3A and phosphorylation of PINK1 is essential for mitophagy^32^ which was decreased in cisplatin-treated HK-2 cells. But in presence of Renogrit (100 µg/ml) their levels increased significantly (p < 0.05) (Fig. 7c,d). This also led to a reduction in the activation of cell death pathway as observed by the significant (p < 0.001) decrease in c-PARP1 levels in Renogrit-treated cells (Fig. 7e). Similar findings were obtained in NAC-treated cells (Fig. 7c–e).

### Renogrit treatment reduced inflammation in human kidney cells

**Figure 8:**
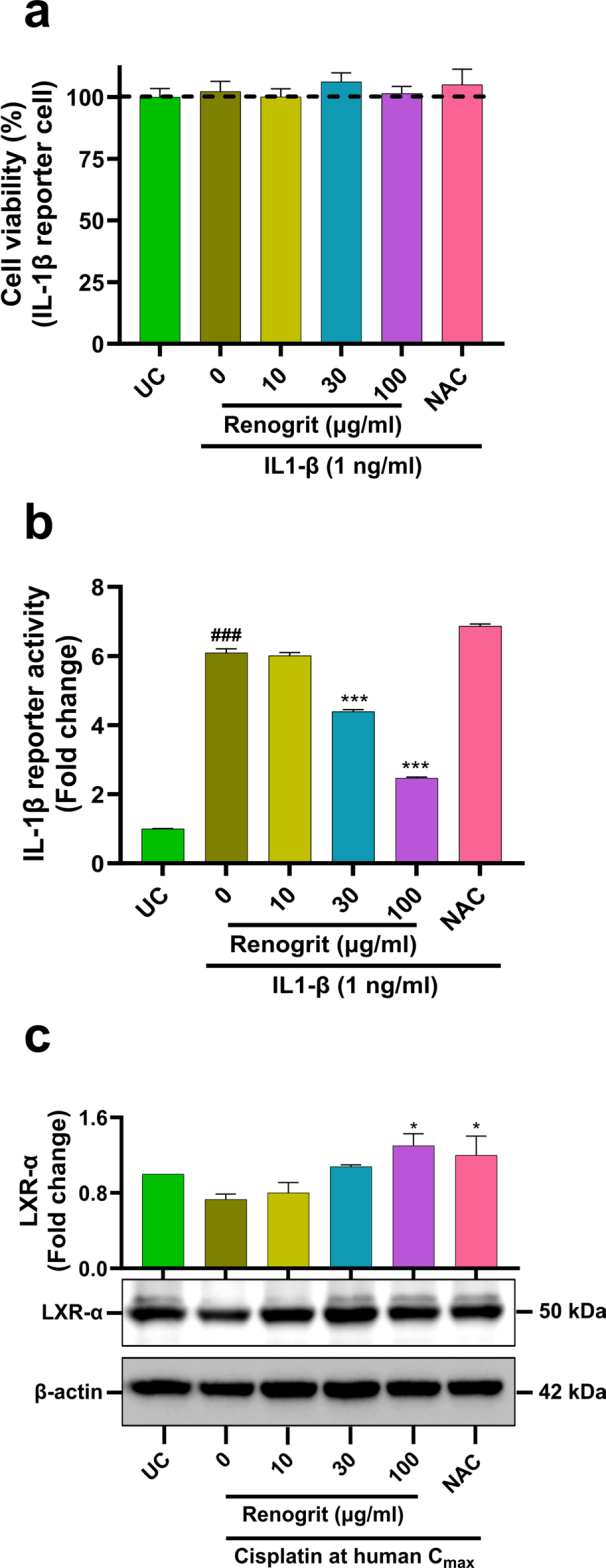
Renogrit reduced inflammation by modulation of IL-1β activity and LXR-α protein levels. (**a**) Renogrit (10–100 µg/ml) treatment showed no effect on the cell viability (%) of IL-1β (1 ng/ml) induced HEK-Blue IL-1β reporter cell. (**b**) Renogrit decreased the IL-1β (1 ng/ml) induced HEK-Blue IL-1β reporter cell activity. (**c**) Representative western blot and relative quantification of LXR-α protein involved in the resolution of cisplatin-induced inflammation showed that Renogrit (10–100 µg/ml) treatment enhanced the protein levels of LXR-α. Data represented as mean ± S.E.M. ^###^ and ***p < 0.001; *p < 0.05.

Pro-inflammatory cytokines, particularly IL-1β is associated with cisplatin-induced kidney injury and inflammation^33^. The activation of LXR-α is known to reduce cisplatin-induced renal inflammation^34^. Hence, in this study the effect of Renogrit on IL-1β and LXR-α was evaluated. HEK-Blue IL-1β reporter cells were used to evaluate the effect of Renogrit against stimulation of IL-1β signaling cascade. To rule out any confounding bias the cytosafety of Renogrit on HEK-Blue IL-1β reporter cells was evaluated. It was observed that Renogrit treatment did not affect the viability of HEK-Blue IL-1β reporter cells (Fig. 8a). Furthermore, in HEK-Blue IL-1β reporter cells stimulated with IL-1β (1 ng/ml), Renogrit (30 and 100 µg/ml) treatment significantly (p < 0.001) reduced the activity of IL-1β which was not observed in NAC (2 mM) treated cells (Fig. 8b). Also, Renogrit (100 µg/ml) treatment on cisplatin-induced HK-2 cells showed a significant (p < 0.05) increase in levels of LXR-α as compared to only cisplatin-treated cells (Fig. 8c). Collectively, Renogrit treatment might ameliorate cisplatin-induced renal injury and inflammation by modulation of IL-1β signalling cascade and LXR-α levels.

### Renogrit treatment did not alter the anticancer property of cisplatin

**Figure 9:**
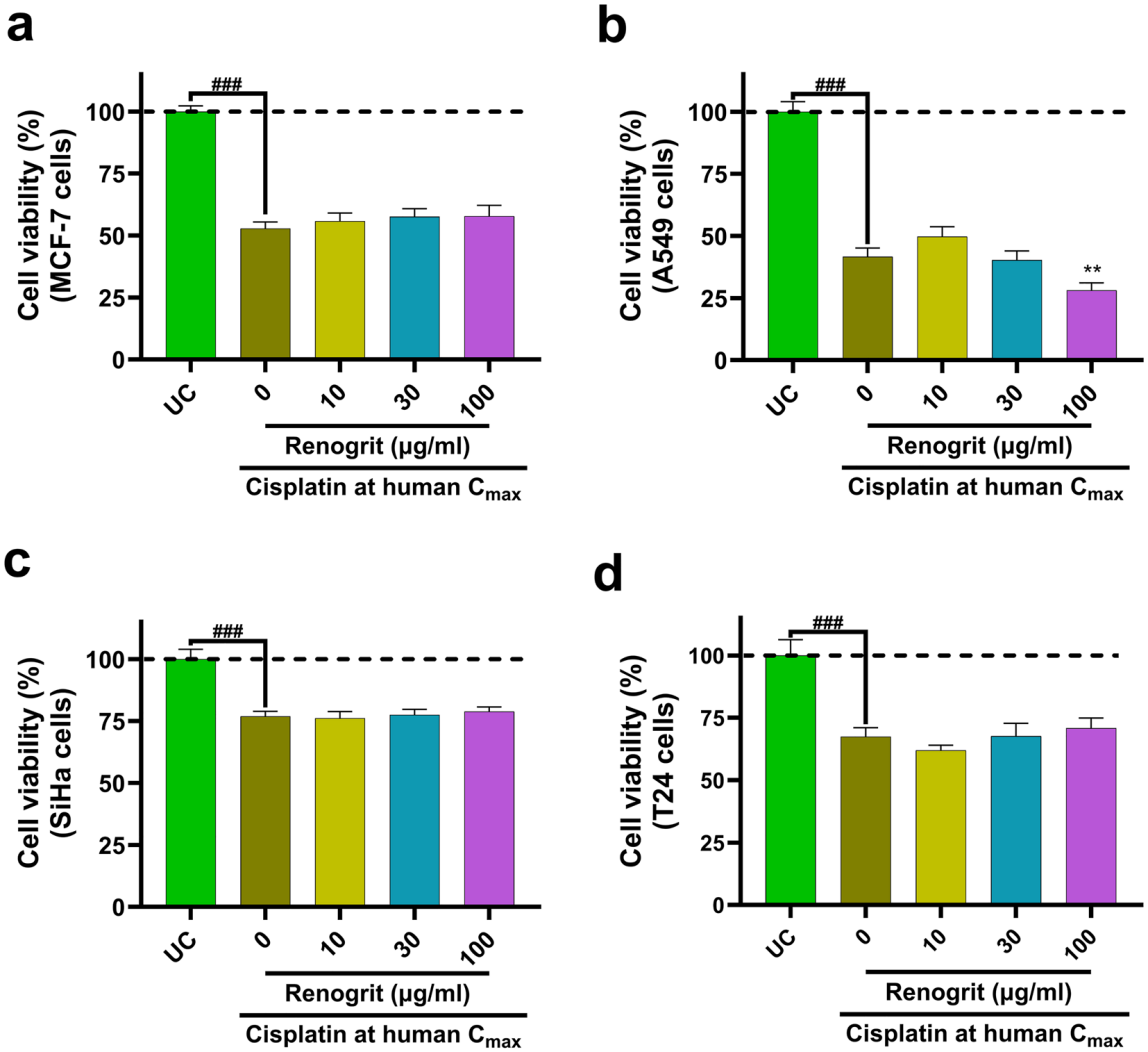
Renogrit did not hindered the anti-cancer effects of cisplatin. Cell viability (%) of (**a**) MCF-7 (human breast cancer cell line), (**b**) A549 (adenocarcinomic human alveolar basal epithelial cells), (**c**) SiHa (human cervical squamous cell carcinoma cell line), and (**d**) T24 (human bladder carcinoma cell line) cells induced with cisplatin at human C_max_ (15 µg/ml) and treated with Renogrit (10–100 µg/ml). Data represented as mean ± S.E.M. ^###^p < 0.001; **p < 0.01.

In order to assess whether the presence of Renogrit hinders the anticancer potential of cisplatin four cancer cells derived from different organs were treated with cisplatin and Renogrit. Although, cisplatin induction significantly (p < 0.001) decreased the viability of MCF-7, A549, SiHa, and T24 cells, but Renogrit did not hinder the anticancer activity of cisplatin (Fig. 9a–d). Interestingly, Renogrit (100 µg/ml) treatment significantly (p < 0.01) decreased the viability of cisplatin-induced A549 cells (Fig. 9b).

### Renogrit treatment decreased toxicity and oxidative stress in N2 and SYS81 strain of *C*.* elegans*

**Figure 10:**
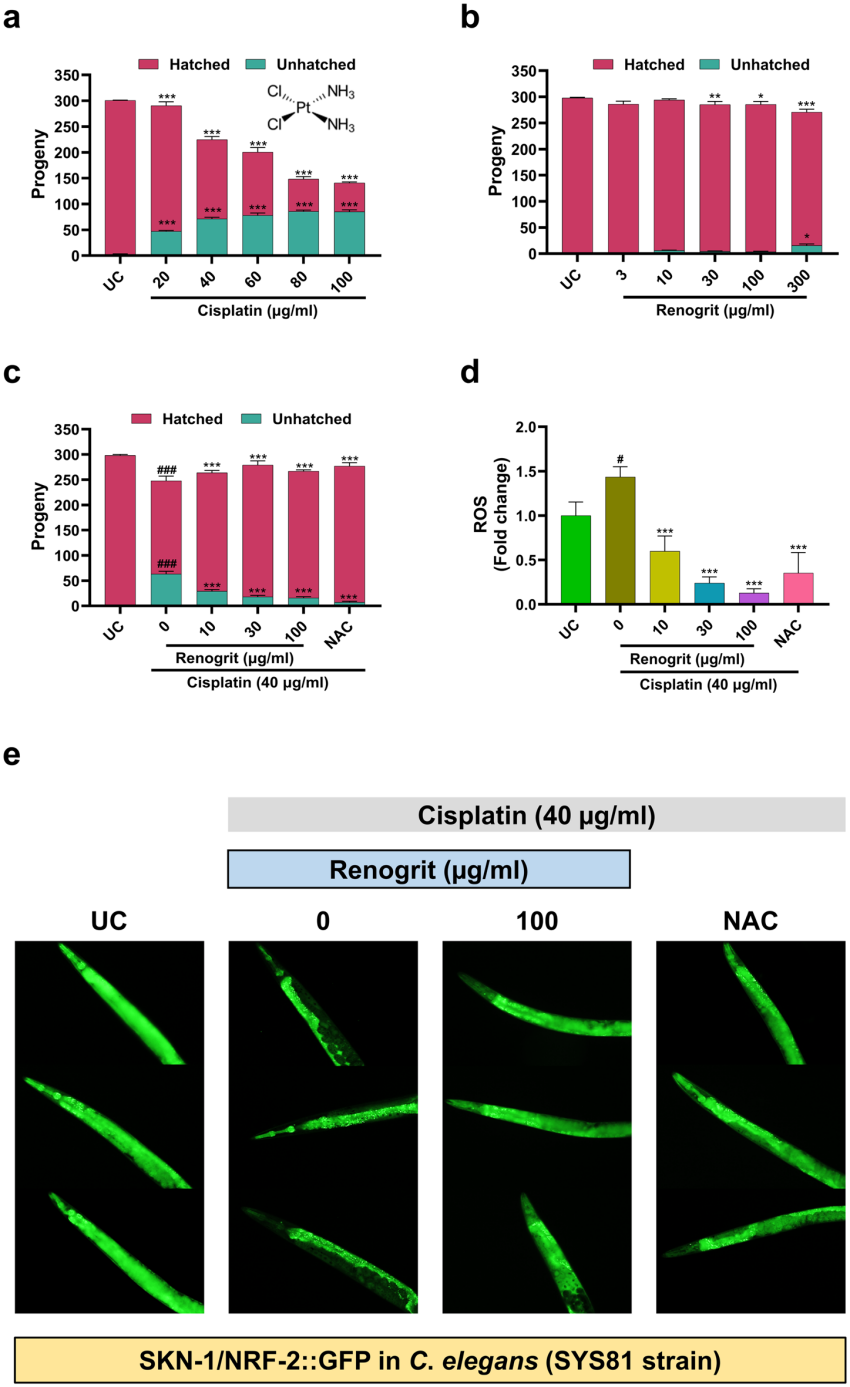
Renogrit decreased cisplatin-induced progeny decline, ROS generation and mitochondrial turnover in *C*. *elegans*. (**a**) Cisplatin (20–100 µg/ml) exposure decreased the progeny (brood size) of *C*. *elegans* (N2 strain). (**b**) Progeny analysis of Renogrit treated *C*. *elegans* (N2 strain). (**c**) Progeny analysis of *C*. *elegans* (N2 strain) induced with cisplatin (40 µg/ml) and treated with Renogrit (10–100 µg/ml) or NAC (4 mM). (**d**) Renogrit reduced the generation of ROS in cisplatin (40 µg/ml) exposed* C*. *elegans* (N2 strain). (**e**) Renogrit decreased nuclear localization (represented as green puncta) of SKN-1/NRF-2 in *C*. *elegans* (SYS81 strain) subjected to cisplatin (40 µg/ml). Magnification = 200 × . Data represented as mean ± S.E.M. ^###^ and ***p < 0.001; ^#^ and *p < 0.05.

Further validations of the pharmacological properties of Renogrit were performed on cisplatin-exposed *C*. *elegans*. Primarily, for the selection of optimum dose for treatment, nematodes were exposed to various concentrations of Renogrit and cisplatin. The progeny in cisplatin (20–100 µg/ml) treated N2 strain of nematode significantly (p < 0.001) decreased (Fig. 10a). Interestingly in Renogrit (300 µg/ml) treated worms a significant (p < 0.05) increase in population of progeny was observed (Fig. 10b). Based on these findings the optimum doses of Renogrit (10, 30, and 100 µg/ml) and cisplatin (40 µg/ml) were selected for further experiments. Upon Renogrit treatment on cisplatin exposed worms, the number of unhatched progenies significantly (p < 0.001) decreased (Fig. 10c). Cisplatin (40 µg/ml) exposure to N2 strain of nematodes led to a significant (p < 0.05) generation of ROS which decreased significantly (p < 0.001) in presence of Renogrit (10, 30, and 100 µg/ml) (Fig. 10d). Furthermore, it was observed that upon cisplatin (40 µg/ml) exposure to SYS81 strain of *C*. *elegans*, SKN-1 (mammalian homologue of NRF2) expression is enhanced which can be observed by the nuclear localization (green puncta formation) compared to untreated control (Fig. 10e). In presence of Renogrit (100 µg/ml) the cisplatin-stimulated SYS81 worms have a similar expression of SKN-1 as untreated control. NAC (4 mM) treatment on the cisplatin-exposed worms also led to comparable findings.

### Renogrit treatment modulated GST-4 and HSP-60 expression in cisplatin-stimulated *C. elegans*

**Figure 11:**
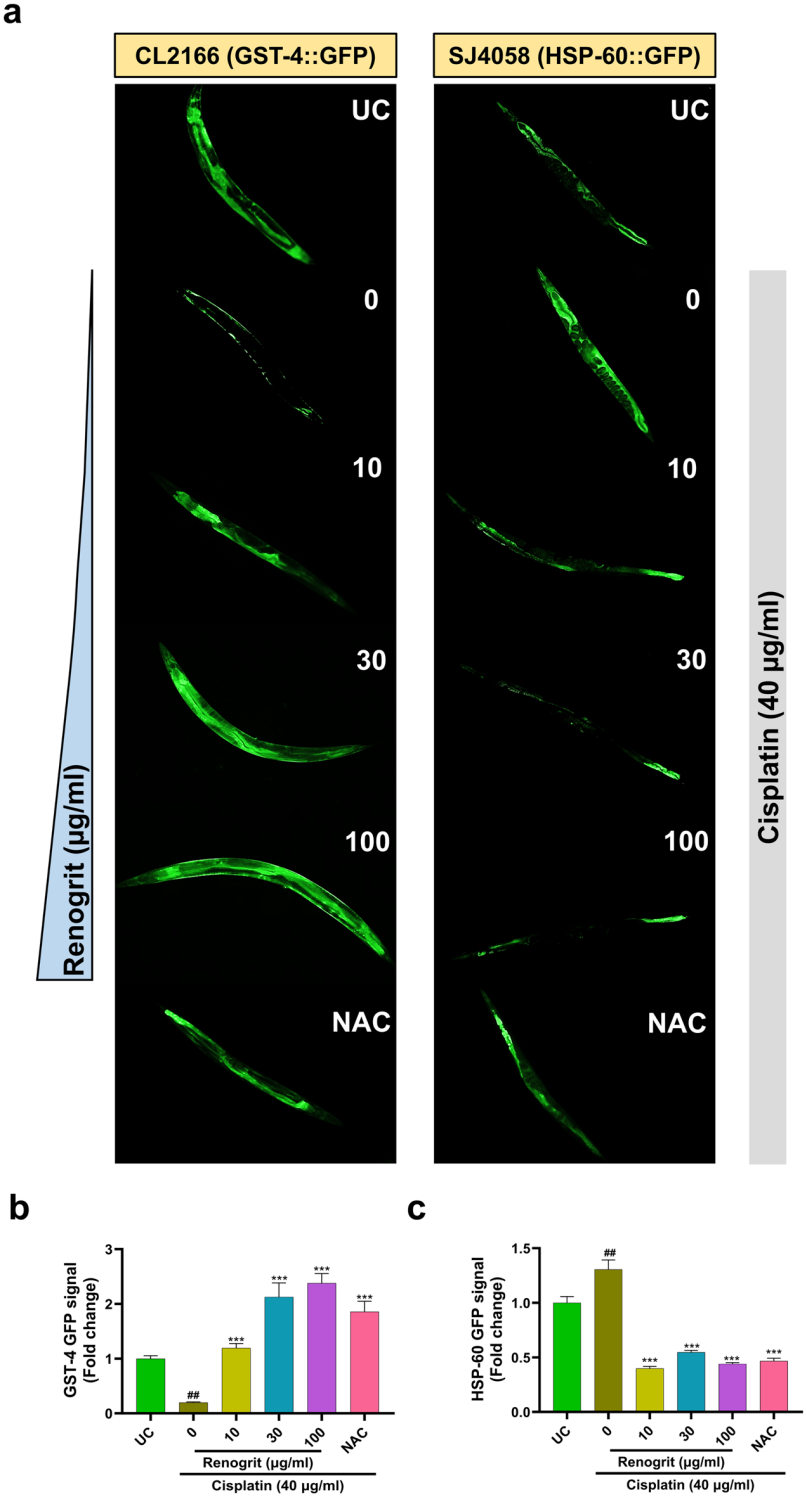
Renogrit decreased cisplatin-induced oxidative stress and mitochondrial aberrations in *C*. *elegans*. (**a**) Representative images of cisplatin (40 µg/ml) exposed CL2166 (GST-4::GFP) and SJ4058 (HSP-60::GFP) strains of *C*. *elegans* showing a decrease in GST-4 and increase in HSP-60 expression respectively. Renogrit (10–100 µg/ml) or NAC (4 mM) treatment normalized the expression of GST-4 and HSP-60 as observed from their respective GFP signals. (**b**) Graph quantifying the relative intensity of GST-4::GFP signal of the treated and untreated worms from their respective images. (**c**) Plate-based quantification of HSP-60::GFP signal intensity in SJ4058 worms after exposure with cisplatin (40 µg/ml) in presence of Renogrit (10–100 µg/ml) or NAC (4 mM). Data represented as mean ± S.E.M. ***p < 0.001; ^##^p < 0.01.

Cisplatin (40 µg/ml) exposure on the CL2166 strain of nematodes led to a significant (p < 0.01) decrease in GST-4 GFP signal. In worms treated with Renogrit (10, 30, and 100 µg/ml) a significant (p < 0.001) increase was observed in GST-4 GFP signal (Fig. 11a,b). Conversely, a significant (p < 0.01) increase in HSP-60 GFP signal was observed in the SJ4058 strain of nematodes exposed to cisplatin (40 µg/ml). This was also apparent from the microscopic observations. In presence of Renogrit (10, 30, and 100 µg/ml) a significant (p < 0.001) decrease was observed in HSP-60 GFP signal (Fig. 11a,c). NAC (4 mM) treated group showed similar observations (Fig. 11a–c).

### Renogrit treatment enhanced lysosome population and mitophagy in cisplatin-exposed *C. elegans*

**Figure 12:**
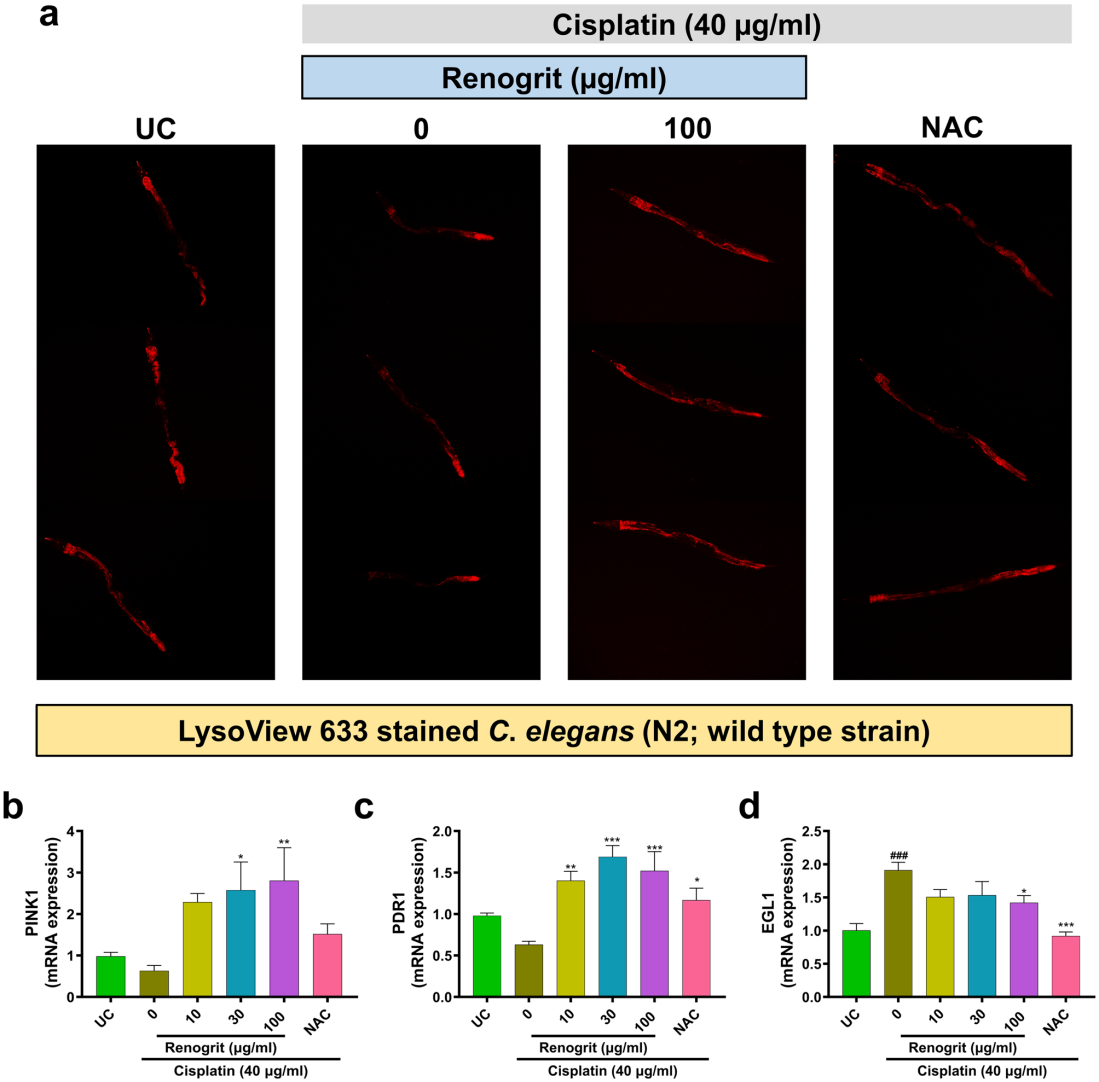
Renogrit modulated cisplatin-induced mitophagy and apoptosis. (**a**) Representative images of LysoView 633 (red) stained *C*. *elegans* (N2 strain) induced with cisplatin (40 µg/ml) and treated with Renogrit (100 µg/ml) or NAC (4 mM). Renogrit treatment enhanced the lysosome number that were decreased due to cisplatin exposure in the nematodes. Renogrit treated *C*. *elegans* (N2 strain) showed increase in mRNA expression of (**b**) PINK1, (**c**) PDR1 genes involved in mitophagy and decrease in pro-apoptotic (**d**) EGL1 mRNA expression. Data represented as mean ± S.E.M. ^###^ and ***p < 0.001; **p < 0.01; *p < 0.05.

Similar to the observed modulations in the lysosome population in vitro, microscopy of cisplatin (40 µg/ml) treated N2 strain of worms showed that the lysosome number has declined as observed from the decrease in levels of LysoView 633 stain. But in the presence of Renogrit (100 µg/ml) the population of lysosomes was comparable to untreated control (Fig. 12a). PINK1 and PDR1 (nematode parkin homologue) are essential for mitophagy and maintenance of mitochondrial turnover^14,18,32^. Renogrit (100 µg/ml) treatment on cisplatin-exposed worms showed that the mRNA expression levels of PINK1 and PDR1 were significantly (p < 0.01) upregulated which declined upon cisplatin exposure (Fig. 12b,c). The mRNA expression levels of EGL1 increased significantly (p < 0.001) in cisplatin-exposed worms but with Renogrit (100 µg/ml) treatment the levels decreased in a significant (p < 0.05) manner. Similar, findings were observed in NAC (4 mM) treated groups.

## Discussion

The current study aimed to analyze the phytopharmacological profile of the Ayurvedic prescription medicine Renogrit using in vitro and *Caenorhabditis elegans* based models. The phytometabolite characterization of Renogrit via HPTLC and UHPLC-based analysis showed the presence of Gallic acid, Bergenin, Methyl Gallate, Quercetin, and Boeravinone B as the major marker compounds. Natural variability in the phytochemical composition of botanical raw materials occurs in response to climate, harvest time, fertilization methods, storage conditions, and processing procedures. The difference in phytochemical composition can lead to variance in bioactivity of the herbal formulation. To ensure optimum bioactivity of Renogrit, three different batches were analyzed for their phytochemical integrity. As observed, the phytochemicals in Renogrit of the three randomly analyzed batches had a similar phytochemical makeup.

NAC was selected as a comparator for the entire study as several non-clinical and a clinical case have been reported wherein NAC was observed to act as a nephroprotective agent in cisplatin treated cell/animal/patient^35–39^. The safety of NAC administration in patients with reduced renal function has also been established^40^. Furthermore, several studies have proposed that NAC has property to modulate oxidative stress, inflammation, apoptosis, and mitophagy^41–44^. Hence, NAC was used as an experimental control for this study.

Prior to the evaluation of bioactivity of Renogrit its effect on viability of HK-2 cells was evaluated. Renogrit was found to be cytosafe at all physiologically relevant concentrations. Moreover, for selection of the clinically relevant dose of cisplatin, its effect on viability of HK-2 was also evaluated. The concentration which showed cytotoxicity in 50% of the cell population and which was nearby its therapeutic concentration (Cisplatin at human C_max_)^24^ was utilized for further in vitro experiments. This allowed us to rule out any confounding bias in our obtained results. After observation of reversal in the cytotoxicity in cisplatin-induced HK-2 cells post Renogrit treatment biomarkers depicting renal tubular injury namely NAG, KIM-1, and NGAL^45^ were also analyzed. It was observed that Renogrit-treated HK-2 cells were able to repel the cytotoxic effects of cisplatin, evident from the normalization of these molecular biomarkers of renal injury. This effect of Renogrit can be co-related with the presence of Gallic acid which was observed to act as a renoprotectant in cisplatin-induced rats^46^.

Oxidative stress and depolarization of mitochondrial membrane are one of the major molecular events linked with the pathophysiological manifestations of cisplatin-induced kidney injury^32^. Herein, when Renogrit was added along with cisplatin on HK-2 cells the generation of ROS was not observed. Further probing of the anti-oxidative properties of Renogrit showed that the activity of the antioxidant enzyme GST that decreased post cisplatin induction also got normalized. Herbal extracts and their isolated phytochemicals are known to enhance GST activity in animal models of cisplatin-induced kidney damage^47,48^. Mitochondria are abundantly present in renal tubular epithelial cells as their function of secretory reabsorption relies on mitochondrial oxidative phosphorylation. Oxidative stress in part promotes mitochondrial depolarization and dysfunction which leads to an imbalance in the normal functionality of renal tubular cells. In the presence of Renogrit, HK-2 cells were able to decrease the adverse effects of cisplatin-stimulated oxidative stress on mitochondrial membrane potential. The phytochemical Bergenin present in Renogrit is known to attenuate renal injury by resisting the change in mitochondrial dynamics, as observed in a rat model^49^.

Cisplatin stimulation is known to trigger several pathways of cell injury which leads to induction of apoptosis and cell death^50^. This is precisely the reason which makes cisplatin an indispensable anti-cancer agent but that also becomes a challenge to protect the healthy cells like normal renal tubular cells from innocent bystander death. Apoptosis is a type of programmed cell death which is mainly mediated by the caspase pathway wherein caspase-3 plays a primary role. Another major cellular event linked with promotion of cisplatin-induced renal tubular cell apoptosis is the activation of MAPK, including ERK (1/2), p38, and JNK^51^. Renogrit treatment decreased the activity and protein levels of the agents involved in cisplatin-induced apoptosis like caspase-3 and phosphorylated MAPKs respectively. Renogrit treatment also decreased the percentage of cells that underwent early apoptosis upon cisplatin stimulation, as observed by Annexin V-7AAD staining. All these renoprotective effects of Renogrit might be due to the presence of Quercetin as one of its major phytometabolites. It has been observed that Quercetin treatment in cisplatin-administered rats decreases the activity of caspase-3 and phosphorylation of ERK (1/2), p38, and JNK in the kidneys^52,53^.

Necroptosis, a form of programmed necrosis, is known to be stimulated in renal tubular epithelial cells. Accumulation of calcium is a major biochemical feature of necroptosis^7,54^. A cascade of kinases namely RIPK-1, RIPK-3, and MLKL are involved in regulation of cisplatin-induced necroptosis in kidney^30^. When HK-2 cells were induced with cisplatin it led to accumulation of calcium which was not the case in Renogrit-treated cells. Furthermore, the overexpression of RIPK-1, RIPK-3, and MLKL mRNA was also normalized in Renogrit-treated cells. Phenolic compounds isolated from herbs are known to reduced necroptosis mediated renal injury post cisplatin administration in mice^13^. As Renogrit is pre-dominantly composed of polyphenolic phytoconstituents it was able to keep the necroptosis activators at bay.

Treatment with high concentrations (≥ 50 µM) of cisplatin leads to a decrease in autophagy which results in the domination of apoptotic cell death in kidney cells^7^. Autophagy can enhance cytoprotection and survival of cisplatin-induced kidney cells. Cisplatin induction in HK-2 cells decreases lysosome biogenesis and autophagy of defective mitochondria (mitophagy)^9^. The process of mitophagy is tightly regulated by PINK1 which in response to loss of mitochondrial membrane potential gets accumulated at the outer membrane of the dysfunctional mitochondria where it gets dimerized, autophosphorylated, and initiates a chain of events to kickstart the process of mitophagy^32^. Cisplatin induction in HK-2 cells is also known to decrease the levels of LC3. Due to its involvement in mitophagy process a decrease in levels of LC3 indicates a defect in the mitophagy machinery^55,56^. A decrease in mitophagy leads to the accumulation of defective mitochondria due to which more ROS is generated causing DNA damage, and apoptosis indicated by cleaved-PARP1^54,57^. Renogrit treatment in cisplatin-induced HK-2 cells normalized lysosome population, enhanced mitophagy, and subsequently prevented apoptosis induced cell death. The phytochemical Quercetin present in Renogrit is known to stimulate mitophagy and prevent cell death in the in vivo models of kidney injury^58,59^.

Calcium flux, mitochondrial damage, and mitophagy dysfunction observed in cisplatin-induced kidney injury have been implicated in the NFκB and NLRP3 inflammasome pathway-mediated release of the pro-inflammatory cytokine IL-1β. An increased expression of IL-1β further triggers the activation of IL-1R inducing activation of NFκB pathway which enhances the release of other inflammatory mediators, exacerbating kidney injury^33,60–62^. The LXR-α present in the kidney is one of the regulators of inflammatory response in cisplatin-induced kidney injury^34^. Renogrit decreased the IL-1β induced NFκB activation as observed from the decreased detection of SEAP released from HEK-Blue IL-1β cells. Also, in cisplatin-induced HK-2 cells, it increased the protein levels of LXR-α. These results suggest that Renogrit has anti-inflammatory properties. This might be due to the presence of several anti-inflammatory phytometabolites in Renogrit^49,63,64^.

In order to use Renogrit to protect from cisplatin-induced kidney injury in clinical settings, it becomes imperative to evaluate whether it affects the anti-cancer potential of cisplatin. Hence, the effect of Renogrit in cisplatin-induced MCF-7 (human breast cancer cell line), A549 (adenocarcinomic human alveolar basal epithelial cells), SiHa (human cervical squamous cell carcinoma cell line), and T24 (human bladder carcinoma cell line) cells were evaluated. It was observed that Renogrit treatment did not hinder the cytotoxic action of cisplatin on these tumor cells. Some previous reports suggest that plant-derived phytochemicals can exert renoprotection without affecting the chemotherapeutic activity of cisplatin on tumors^53,55,65,66^. Moreover, it was observed that in A549 cells, Renogrit enhanced the cytotoxic potential of cisplatin in a concentration-dependent manner. This effect of Renogrit can be attributed to the presence of *Boerhavia diffusa* L. extract as it is known to decrease cell viability of A549 cells^21^.

The effects of Renogrit were further evaluated in the in vivo model of *C*. *elegans* exposed to cisplatin. Initially, a suitable dose of cisplatin and Renogrit was screened based upon the decline in the progeny of worms. Upon selection of the suitable dose, the effects of Renogrit on cisplatin-stimulated worms were evaluated. It was found that the decrease in progeny mediated by cisplatin was reversed in response to Renogrit treatment in a dose-dependent manner. Furthermore, Renogrit treatment also perturbed the generation of ROS in worms exposed to cisplatin. Plant-based medicines are known to decrease defects in the growth and reproduction stages of nematodes, majorly in response to their antioxidant properties^67^. As expected we also found a decline in ROS generated due to Renogrit treatment in worms exposed to cisplatin. The NRF2 is activated under oxidative stress and inhibition of mitophagy in mammalian cells. Similarly, nematode homologue of NRF2, SKN-1 (SKiNhead-1) is activated during oxidative stress and inhibition of mitophagy^18,68^. Renogrit treatment reduced the activation of SKN-1 induced by cisplatin exposed SYS81 strain of *C*. *elegans* as observed from the decrease in nuclear localization of SKN-1 GFP signal in their intestine. Hence, it can be stated that Renogrit-treated worms were able to ward off oxidative stress and maintain mitochondrial turnover.

The GST-4 in *C*. *elegans* shares a sequence similarity with the GST family in humans. Overexpression of GST-4 is known to increase the stress resistance in the nematodes^69^. Renogrit treated CL2166 strain of worms showed high expression of GST-4 upon exposure of cisplatin as observed by increase in the GST-4 GFP signal. This was also observed in the in vitro findings wherein the GST levels were increased in the HK-2 cells treated with Renogrit. High levels of ROS can directly oxidize mitochondrial proteins and promote protein aggregation which leads to the accumulation of the mitochondrial chaperone, HSP-60 in the mitochondria^70^. A preclinical study by Timurkaan M et al.^71^ suggests that in cisplatin-treated rats the levels of HSP-60 get upregulated in response to oxidative stress. Furthermore, they found that rats treated with the flavonoid epigallocatechin gallate decreased the protein expression of HSP-60 in the rats injected with cisplatin. Parallel to these observations when the worms of strain SJ4058 were exposed to cisplatin an increase in HSP-60 GFP signal was observed which was found to decrease in a dose-dependent manner in worms co-treated with Renogrit. These results suggest that Renogrit treatment prevents cisplatin-induced oxidative stress and subsequent mitochondrial dysfunction.

Another important mechanistic aspect that was similar to the in vitro findings on cisplatin-induced HK-2 cells was the decrease in lysosome population in the N2 strain of *C. elegans*. The number of lysosomes in nematodes decreased in response to cisplatin exposure as observed by LysoView 633 (pH sensitive) stained nematodes. Also, the genes involved in mitophagy namely PINK1 and PDR1 (Homologue of mammalian Parkin) were also found to decrease due to cisplatin exposure. Renogrit co-treated *C*. *elegans* were able to maintain a normal population of lysosomes and also showed an increase in genes responsible for mitophagy. Cisplatin is known to induce the overexpression of EGL-1 gene, the nematode homologue of mammalian pro-apoptotic Bcl-2 family members^72,73^. Renogrit treatment subdued the EGL-1 gene overexpression which signals that the pro-apoptotic signals generated in cells of *C*. *elegans* have been reduced.

**Figure 13:**
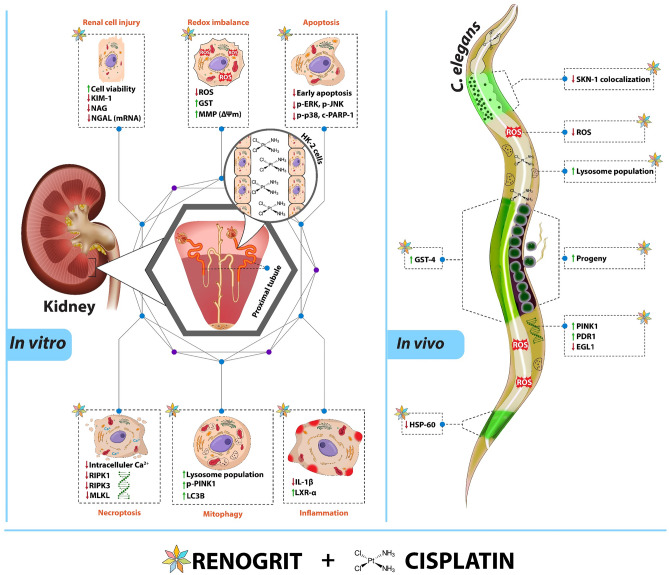
Representation of the molecular changes induced by cisplatin in HK-2 cells and in *C*. *elegans* along with the subsequent therapeutic modulation by Renogrit.

In conclusion, the validations of the mechanistic aspects of Renogrit were performed in HK-2 cells and various strains of *C*. *elegans*. Renogrit treatment decreases the renal tubular cell injury by mitigating cisplatin-induced oxidative stress, mitochondrial dysfunction, apoptosis, necroptosis, mitophagy, and inflammation by targeting multiple pathways of cell injury without affecting the anti-cancer potential of cisplatin. Similarly, in *C*. *elegans* the defects induced by cisplatin namely decrease in progeny, increase in oxidative stress, defects in mitochondria, and lowered mitophagy were mitigated by Renogrit. A summary representation of the current study is depicted in Fig. 13. Taken together, the study suggests that Renogrit has clinically relevant properties for the management of cisplatin-induced nephrotoxicity.

## Material and methods

### Reagents

Several batches of Renogrit (Laboratory internal code # CHIH/RENA/0222/2304, CHIH/RENA/0222/2321, CHIH/RENA/0322/2431) were sourced from Divya Pharmacy, India. Cisplatin (CSI1934B) was obtained from GLS Pharma Ltd., India. Dulbecco's Modified Eagle Medium (DMEM) (D2902), McCoy’s 5A medium (M4892), and Nutrient Mixture F-12 Ham (N6760) were obtained from Sigma-Aldrich, USA. Antibiotic–antimycotic solution (A002A), FBS (RM9955), and Alamar blue (TC235) were procured from HiMedia, India. N-acetyl-L-Cysteine (NAC) (47866) and 4-Nitrophenyl-N-acetyl-β-D-glucosaminide (72565) were obtained from SRL, India. Human KIM-1 ELISA kit (ITLK02287) was obtained from GBiosciences, USA. Fluo-4 AM (F14201), Verso cDNA synthesis kit (AB-1453/A), and PowerUp SYBR Green Master Mix (A25742) were procured from Thermo Fisher Scientific, USA. 2′, 7′-Dichlorofluorescin diacetate (H_2_DCFDA) (D6883) was obtained from Sigma-Aldrich, USA. Reagents Z-DEVD-AMC (13421) and JC-1 (22200) were purchased from AAT Bioquest, USA. The FITC Annexin V Apoptosis Detection kit with 7-AAD (640922) was obtained from BioLegend, USA. Recombinant Human IL-1β (200-01B) was purchased from PeproTech, USA. LysoView 633 (70058) was procured from Biotium, USA. HEK-Blue IL-1β reporter cells (hkb-il1bv2) were procured from InvivoGen, USA.

### Phytochemical analysis of Renogrit

Renogrit (500 mg) powder (batch# CHIH/RENA/0322/2431) was dissolved in 10 ml methanol: water (80: 20 v/v), sonicated for 20 min, and centrifuged at 6000 rpm for 10 min by Sorvall ST-8R (Thermo Fisher Scientific, USA) and filtered using 0.45 µm nylon filter. An appropriate quantity of each standard was dissolved in methanol to prepare a stock solution of 1000 μg/ml from which working standards of Gallic acid (GA: 100 μg/ml), Methyl gallate (MG: 100 μg/ml), Quercetin (QT: 20 μg/ml) and Bergenin (BG: 100 μg/ml) were prepared. A HPTLC system (CAMAG, Switzerland) equipped with an automatic TLC sampler (ATS4), TLC scanner 4, TLC Visualizer, and integrated software winCATS (Version 1.4.10) was used for the HPTLC analysis on a pre-coated silica gel 60 F254 aluminium backed TLC plate. For fingerprint analysis 10 μl of each standard and 4 μl of sample were applied as 8 mm band using spray-on technique on a TLC plate. The plate was developed using CAMAG twin trough chamber pre-saturated (10 min) with mobile phase (Toluene: ethyl acetate: methanol: formic acid (5:4:1:0.2 v/v/v/v). TLC plate was developed up to 70 mm, dried under warm air, and visualized under UV 254 nm. The image was documented and the same TLC was scanned at 280 nm, slit dimension was 6 × 0.45 mm, scanning speed was 20 mm/s, with data resolution of 100 μm/step. A deuterium lamp was used under absorption mode. For quantification, several concentrations of each standard solution were applied on the plate and on the basis of linearity plot concentration of specific phytochemical, concentration of the same phytochemical in Renogrit was evaluated under same chromatographic conditions.

In order to rule out any deviations in the quantity of phytometabolites characterized by HPTLC, further analysis of phytometabolites was performed by UHPLC. Three different batches of Renogrit were evaluated to generate a thorough quantitative phytochemical profile. UHPLC-based analysis of phytocompounds was evaluated on Prominence-XR UHPLC system (Shimadzu, Japan) fitted with Quaternary pump (Nexera XR LC-20AD XR), diode array detector (DAD SPD-M20 A), auto-sampler (Nexera XR SIL-20 AC XR), degassing unit (DGU-20A 5R) and column oven (CTO-10 AS VP). Separation was achieved using a Shodex C18-4E (5 µm, 4.6 × 250 mm) column subjected to binary gradient elution. The two solvents used for the analysis consisted of water comprising 0.1% acetic acid (solvent A) and acetonitrile (solvent B). Gradient programming of the solvent system was primarily at 0–5% B for 0–10 min, 5–10% B from 10 to 20 min, 10–20% B from 20 to 30 min, 20–30% B from 30 to 40 min, 30–50% B from 40 to 50 min, 50–70% B from 50 to 60 min, 70–90% B from 60 to 70 min, 90–0% B from 70 to 72 min, 0% B from 72 to 75 min with a flow rate of 1 ml/min. 10 µl of standard and test solution were injected, the column oven temperature was maintained at 35 °C, and the detector wavelength was set at 270 nm throughout the analysis. Data were presented as mean ± S.E.M (n = 3).

### Cell culture and treatments

HK-2 cells (CRL-2190) were procured from ATCC, USA. Cells were grown with DMEM/ Nutrient Mixture F-12 Ham containing 5% FBS and 1% antibiotic–antimycotic solution. MCF-7 (human breast cancer cell line), A549 (adenocarcinomic human alveolar basal epithelial cells), SiHa (human cervical squamous cell carcinoma cell line), and T24 (human bladder carcinoma cell line) were procured from the ATCC licensed repository, National Centre for Cell Science, India. MCF-7, A549, and SiHa were propagated in DMEM supplemented with 10% FBS and 1% antibiotic–antimycotic solution. T24 cells were cultured in McCoy’s 5A medium containing 10% FBS and 1% antibiotic–antimycotic solution. The HEK-Blue IL-1β reporter cells were cultured as per the manufacturer’s instructions. All the cells were used within 4–6 passages post 70–80% cell confluency. Unless specified otherwise, all cells were pre-treated for 24 h with Renogrit and then co-treated with cisplatin and Renogrit for 24 h. For evaluation of the biological effects of Renogrit the concentration of cisplatin did not exceed 15 µg/ml (≈ 50 µM) which is relevant to the maximum observed blood plasma concentration of cisplatin (C_max_ of cisplatin) in patients after drug treatment^24^. So, the dose of 15 µg/ml was considered as Cisplatin at human C_max_. Renogrit (batch# CHIH/RENA/0322/2431) was used for pharmacological evaluation. The concentration of positive control (NAC) used was 2 mM in line with the previous in vitro studies of cisplatin-induced nephrotoxicity^74^. The untreated control (UC) group was taken as the normal control.

### Cell viability

HK-2 cells were seeded at a density of 10,000 cells/well in a 96-well plate. The effect of Renogrit (3–300 µg/ml) and cisplatin (5–25 µg/ml) on the viability of HK-2 cells was evaluated post 24 h of incubation by Alamar blue dye. Similarly, recovery of cell viability in response to Renogrit (3–100 µg/ml) or NAC in cisplatin (15 µg/ml) induced HK-2 was detected. After incubation with Alamar blue dye, the plates were read at Ex. 560/ Em. 590 nm on EnVision multimode plate reader (PerkinElmer, USA). Data were presented as mean ± S.E.M (n = 3).

### Evaluation of NAG and KIM-1

The supernatants of HK-2 cells were used for NAG and KIM-1 estimation after treatment with Renogrit (10, 30, and 100 µg/ml) or NAC along with the human C_max_ of cisplatin. NAG evaluation assay was performed using 4-Nitrophenyl-N-Acetyl-B-D-Glucosaminide as the substrate^75^. The final enzyme activity values were normalized with protein concentrations estimated by the BCA method. KIM-1 estimation was done using a Human KIM-1 ELISA kit as per the manufacturer’s instructions. Data were presented as mean ± S.E.M (n = 3).

### Assessment of ROS generation and GST activity

HK-2 cells were seeded at a density of 10,000/well in a 96-well plate and incubated overnight. The next day, cells were washed twice with DPBS and stained with 10 µM H_2_DCFDA. After 30 min of incubation at 37 °C, cells were again washed with PBS and simultaneously loaded with 2 × concentrations of Renogrit (10, 30, and 100 µg/ml) or NAC along with the human C_max_ of cisplatin post which readings were immediately taken at Ex. 485/Em. 535 nm by EnVision multimode plate reader (PerkinElmer, USA) in kinetics mode for 2 h. The difference in the final and initial fluorescence measurements taken up to 120 min was plotted. The GST activity was measured by using 1-chloro-2,4-dinitrobenzene (CDNB) as a substrate^76^. Data were presented as mean ± S.E.M (n = 3).

### Evaluation of mitochondrial membrane potential

The use of JC-1 stain was done for the determination of mitochondrial membrane potential of HK-2 cells treated with Renogrit (10, 30, and100 µg/ml) or NAC along with the human C_max_ of cisplatin. Ratiometric analysis of JC-1 aggregates was performed as per the manufacturer’s instructions. Data were presented as mean ± S.E.M (n = 3).

### Apoptosis assay

Determination of Apoptosis was done by FITC Annexin V Apoptosis Detection kit with 7-AAD as per the manufacturer’s instruction. Acquisition was done by Attune NxT Flow Cytometer (Invitrogen, USA). Data were presented as mean ± S.E.M (n = 3).

### Evaluation of caspase-3 activity

Z-DEVD-AMC a fluorogenic substrate was used for estimation of caspase-3 activity in lysates of treated cells as per the manufacturer’s instructions. The final values were normalized with protein concentrations. Data were presented as mean ± S.E.M (n = 3).

### Qualitative evaluation of intracellular calcium levels

The calcium indicator Fluo-4, AM was used for the microscopy-based evaluation of intracellular levels of calcium in HK-2 cells treated with Renogrit (100 µg/ml) or NAC along with the human C_max_ of cisplatin. Briefly, post-treatment cells were washed with DPBS and incubated with 3 µM Fluo-4, AM for detection of calcium and Hoechst 33342 (1:1000) for 30 min. Post-incubation cells were washed with DPBS and imaged on ImageXpress Pico (Molecular Devices, USA) using DAPI and FITC filters at 100 × magnification. A total of 15 images were captured per treatment.

### Evaluation of lysosome population

The change in lysosome number in response to treatment with Renogrit (10, 30, and 100 µg/ml) or NAC along with the human C_max_ of cisplatin was detected by flow cytometry upon staining the cells with LysoView 633 (1:1000) for 30 min. The acquisition was done in Attune NxT Flow Cytometer (Invitrogen, USA). Data were presented as mean ± S.E.M (n = 3).

### Quantification of proteins by western blot

Cells were lysed with cold RIPA buffer containing (100 mM Tris, 150 mM NaCl, 1 mM EGTA, 1 mM EDTA 0.5% sodium deoxycholate, 1% Triton X-100, and freshly supplemented with protease inhibitor (A32963, Thermo Fisher Scientific) and PhosSTOP phosphatase inhibitor (4906845001, Roche) as per manufacturer’s protocol. Protein concentration in lysates was estimated by BCA method. The lysates containing 20–25 µg proteins were resolved in 10%—12% SDS-PAGE followed by transfer to a PVDF membrane (1620177, Bio-Rad). Primary antibodies used for evaluation are mentioned in Table 2. The secondary antibodies were obtained from Invitrogen, USA. Incubation time and antibody concentration were followed as per the manufacturer’s protocol. Protein bands were developed using Enhanced chemiluminescent HRP substrate (WBKLS0500, Millipore, USA) in an ImageQuant LAS 500 (GE Healthcare, USA) instrument, and blots were further processed and quantified using Image Quant TL version 8.2 software (GE Healthcare, USA) and calculated according to reference bands of β-actin.

**Table 2:** List of antibodies used for western blotting. All these antibodies were obtained from Invitrogen, USA except for NR1H3 (LXR-α) which was procured from Novus Biologicals, USA.

Antibody	Catalog number	Lot number
Cleaved PARP-1	44-698G	2347845
NR1H3 (LXR-α)	H00010062-M08	MA131-3E9
ERK1/ERK2	13-6200	UG286805
p-ERK1/p-ERK1	36-8800	VB2933212
JNK1/JNK2	AHO1362	RA23154
p-JNK1/p-JNK2	44-682G	2259453
p38	MA5-15116	WA3165876
p-p38	44-684G	2249202
LC3A/LC3B	PA1-16931	WF3308803A
p-PINK-1	PA5-105356	YD3880896A
β-actin	MA5-11869	XE3585078

### Evaluation of IL-1β activity

HEK-Blue IL-1β reporter cells co-treated with IL-1β (1 ng/ml) and Renogrit (10, 30, and 100 µg/ml) or NAC and incubated for 24 h. Post incubation the supernatant was collected and the secreted embryonic alkaline phosphatase (SEAP) levels were evaluated by QUANTI-Blue as per the manufacturer’s instructions. The optical density was read at 630 nm using EnVision multimode plate reader (PerkinElmer, USA). Data were presented as mean ± S.E.M (n = 3).

### Cell viability analysis of cisplatin-induced and Renogrit treated cancer cells

MCF-7, A549, SiHa, and T24 cells were seeded at a density of 10,000 cells/ well in a 96-well plate. Post-treatment the cell viability was assessed using Alamar blue dye as mentioned before. Data were presented as mean ± S.E.M (n = 3).

### Nematode strains and general methods

*Caenorhabditis elegans* strains were obtained from the Caenorhabditis Genetics Center at the University of Minnesota, USA, and maintained as described previously^77^. The N2 (Bristol) strain was used as wild-type in all experiments and the following alleles and transgenic strains were used in this study: CL2166 (GST-4::GFP reporter strain)^78^, SJ4058 (HSP-60::GFP reporter strain)^79^, and SYS81 (SKN-1::GFP reporter strain)^80^.

### Nematode brood size assessment and treatment procedure

The hatched eggs released L1 larvae which were used for exposure to various treatments. The L1 nematodes were exposed to different concentrations of Cisplatin (20–100 μg/ml) or Renogrit (3–300 μg/ml). A single L3 stage worm of each condition was isolated onto fresh petriplates with *E. coli* OP50. Worms were transferred to fresh plates with Cisplatin and Renogrit every other day. The progeny and number of oocytes laid were counted after 3 days. Parent worms were removed and progeny were allowed to develop for 48 h before evaluation of worms. Cisplatin (40 μg/ml) and Renogrit (10, 30, and 100 μg/ml) or NAC (4 mM) were used for further experimentation. Unless specified otherwise, L1 nematodes were pre-treated with Renogrit (10, 30, and 100 μg/ml) or NAC (4 mM) for 24 h after which the nematodes were transferred to NGM plate seeded with *E. coli* OP50 with or without 40 μg/ml of cisplatin exposure along with Renogrit or NAC for 4 days. Counting was done using the ZEISS Stemi 305 stereo microscope (Carl Zeiss, Germany). Data were presented as mean ± SEM (n = 5).

### Measurement of ROS Levels in N2 strain of *C*. *elegans*

ROS levels in Renogrit and cisplatin-exposed *C. elegans* were detected by H_2_DCFDA dye as previously described^67^ with slight modifications. Briefly, L3 stage N2 worms were stained with H_2_DCFDA (200 µM) for 2 h after which worms were washed twice with M9 buffer and resuspended. 200 µl of worm suspension was transferred to a 48-well plate with simultaneously loaded with 2 × concentrations of Renogrit (10, 30, and 100 µg/ml) or NAC along with cisplatin (40 µg/ml). Fluorescence measurements were immediately commenced and taken till 24 h at Ex. 485/Em. 535 nm by EnVision multimode plate reader (PerkinElmer, USA). The change in fluorescence was plotted as mean ± SEM (n = 3).

### Assessment of SKN-1 expression in SYS81 strain of* C. elegans*

To analyze SKN-1/NRF2 expression, nematodes of SKN-1∷GFP reporter strain were treated as described before and then processed for microscopy. Images were acquired using a FITC filter on an Olympus BX43 microscope equipped with a Mantra imaging platform (PerkinElmer, USA) and further processed on the Inform 2.2 software suite (PerkinElmer, USA). A total of 10 images of worms from different progenies were evaluated for deriving the conclusion.

### Assessment of GST-4 GFP signal in CL2166 strain of* C. elegans*

For measurement of GST-4 induction, nematodes of GST-4∷GFP reporter strain were treated and then processed for microscopy as described before. The GFP images were captured and fluorescence intensity was measured using ImageJ software (NIH, USA). Data were presented as mean ± SEM (n = 10).

### Assessment of HSP-60 GFP signal in SJ4058 strain of *C. elegans*

For evaluation of HSP-60 expression, nematodes of the HSP-60∷GFP reporter strain were treated and then processed for microscopy as described before. Separately, a plate-based assay was also performed where treated worms were transferred to a 96-black well plate and measured for fluorescence at Ex. 480/ Em. 510 nm on infinite 200Pro (Tecan, Switzerland) plate reader. The worms were lysed and protein concentration was determined using the BCA method. The obtained values were further normalized with protein concentration. Data were presented as mean ± SEM (n = 3).

### Quantification of mRNA expression in HK-2 cells and *C*. *elegans* using quantitative real-time PCR (qRT-PCR)

The mRNA expression analysis was performed as previously described^77^. Primers used for the study are mentioned in Table 3. Data were analysed by using fold changes in relative mRNA expression (2^−ΔΔCt^) as compared to control using housekeeping gene β-actin and ACT-1, for HK-2 cells and *C*. *elegans,* respectively. Data were presented as mean ± S.E.M (n = 3).

**Table 3:** Sequences of primers used for qRT-PCR of HK-2 cells (H) and *C*. *elegans* (CE).

H-RIPK3	Forward	ATGTCGTGCGTCAAGTTATGG
	Reverse	CGTAGCCCCACTTCCTATGTTG
H-RIPK1	Forward	GGGAAGGTGTCTCTGTGTTTC
	Reverse	CCTCGTTGTGCTCAATGCAG
H-MLKL	Forward	AGGAGGCTAATGGGGAGATAGA
	Reverse	TGGCTTGCTGTTAGAAACCTG
H-NGAL	Forward	GAAGTGTGACTACTGGATCAGGA
	Reverse	ACCACTCGGACGAGGTAACT
H-β-actin	Forward	CACCAACTGGGACGACAT
	Reverse	ACAGCCTGGATAGCAACG
CE-EGL1	Forward	CTAGCAGCAATGTGCGATGAC
	Reverse	GGAAGCATGGGCCGAGTAG
CE-PDR1	Forward	GACTACAAGGTGATCTCAGCGA
	Reverse	CGTGGCATTTTGGGCATCTT
CE-PINK1	Forward	CAAGGCGAGCCTGAAAGGA
	Reverse	GCCGAGAATATTTCCCGCCA
CE-ACT-1	Forward	ACGACGAGTCCGGCCCATCC
	Reverse	GAAAGCTGGTGGTGACGATGGTT

### Data analysis

Statistical analysis was performed using GraphPad Prism 10 (GraphPad Software, USA). Data were presented as mean ± S.E.M. Significance of the difference between different treatment groups was determined by one-way and two-way ANOVA followed by Dunnett’s post-hoc analysis. The results were considered to be statistically significant at p < 0.05.

### Supplementary Information

Supplementary Figures.

## Data Availability

Data are available on request from the corresponding author.
