# Supplementary material for: Renogrit selectively protects against cisplatin-induced injury in human renal tubular cells and in Caenorhabditis elegans by harmonizing apoptosis and mitophagy
Source: Sci Rep. 2024 Aug 21;14:19443. doi: 10.1038/s41598-024-69797-3 (PMC11339073; doi:10.1038/s41598-024-69797-3)

Supplementary figures

Renogrit Selectively Protects Against Cisplatin-induced Injury in Human Renal Tubular Cells and in *Caenorhabditis elegans* by Harmonizing Apoptosis and Mitophagy

Acharya Balkrishna, Vivek Gohel, Nishit Pathak, Monali Joshi, Rani Singh, Ankita Kumari, Rishabh Dev, and Anurag Varshney*
***Corresponding author: Anurag Varshney**
E-mail address: anurag@patanjali.res.in, anurag@prft.co.in

Full length blot of Fig. 5d (p-ERK, Total ERK, and β-actin)

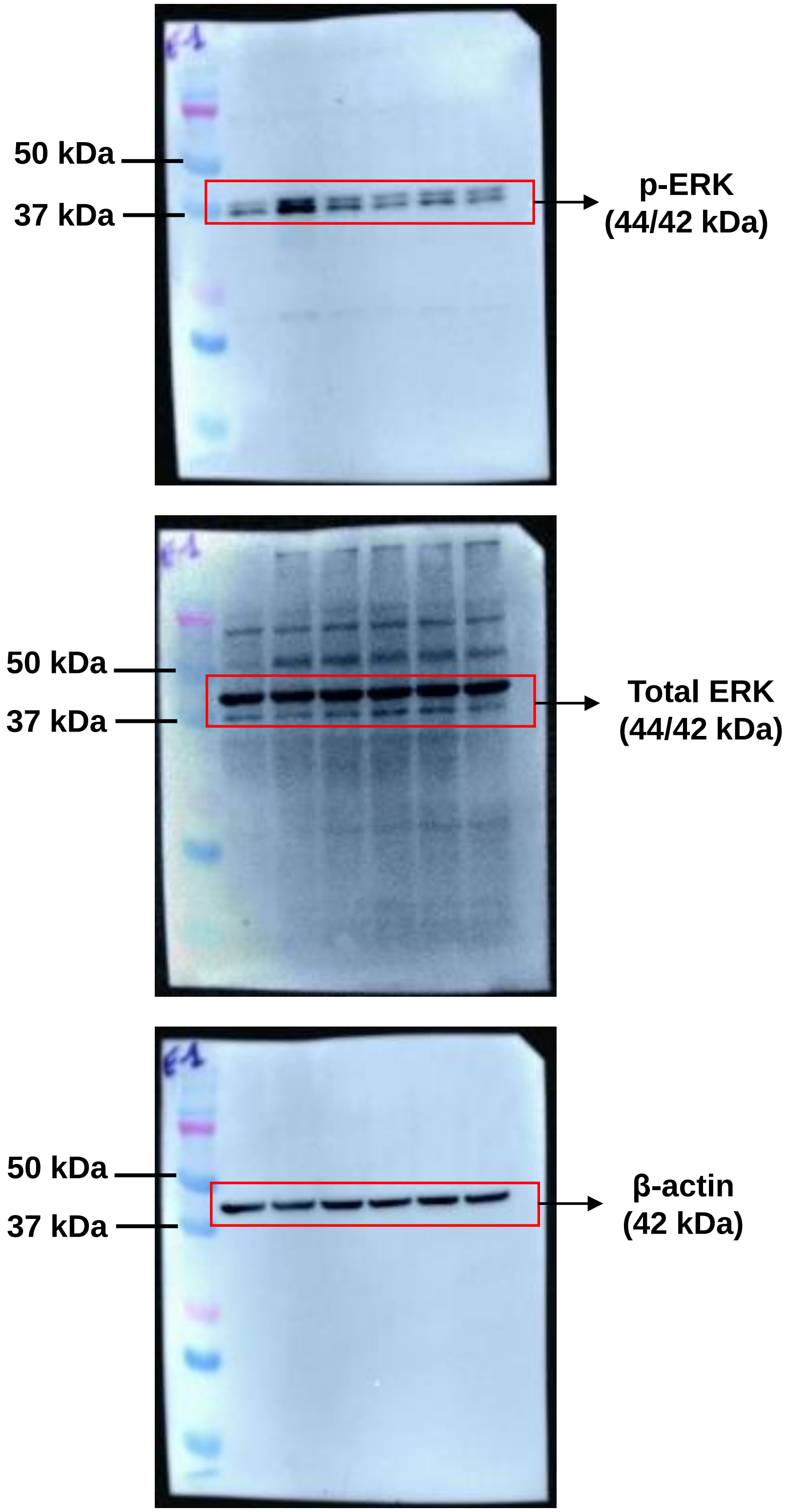

Full length blot of Fig. 5e (p-JNK, Total JNK, and β-actin)

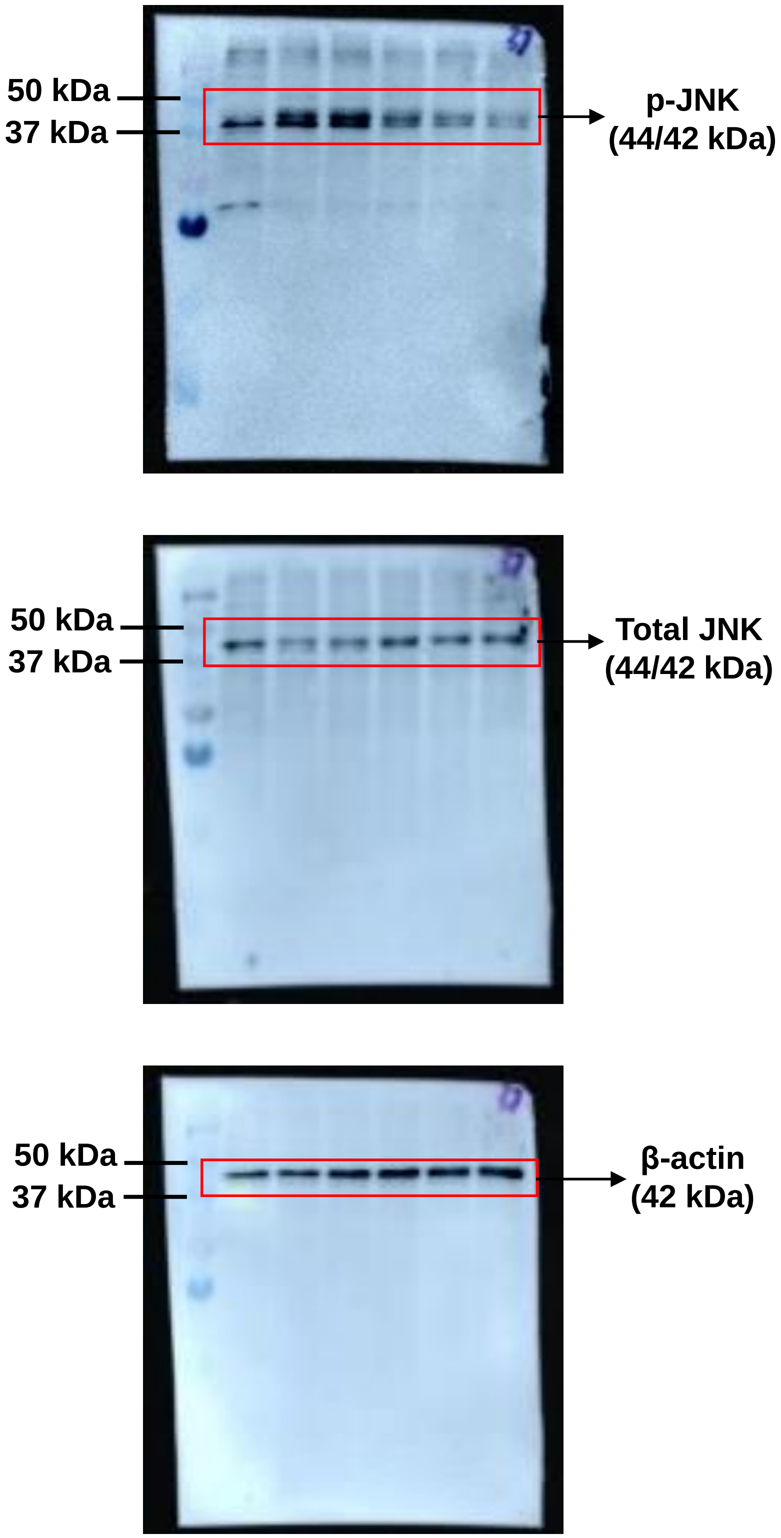

Full length blot of Fig. 5f (p-p38, Total p38, and β-actin)

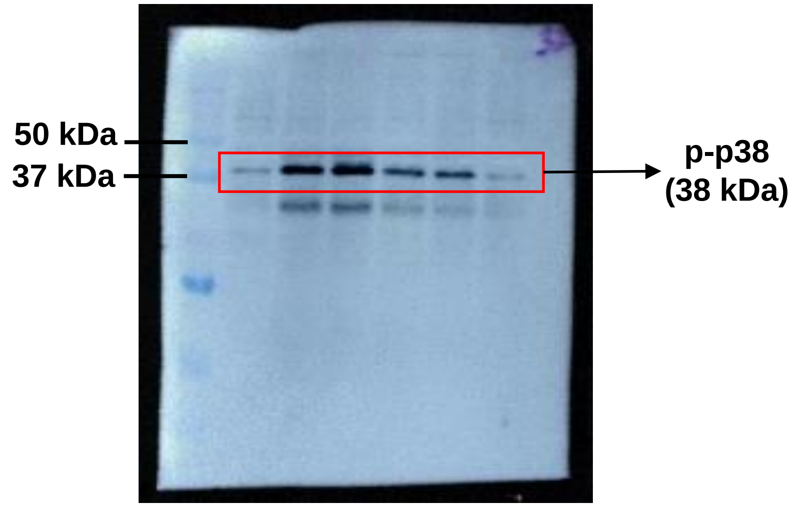

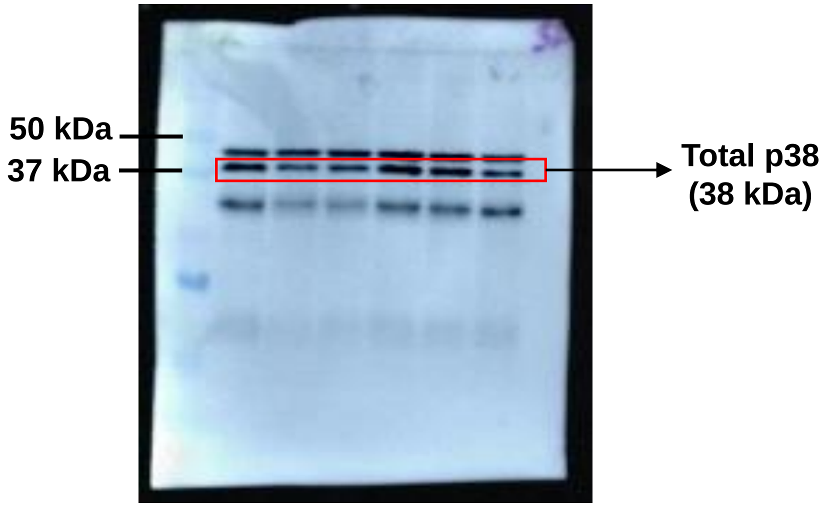

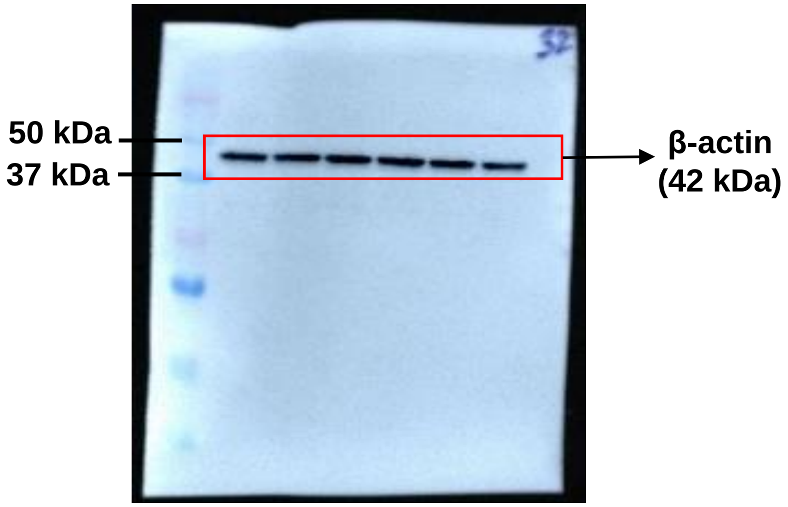

Full length blot of Fig. 7c (LC3A, LC3B, and β-actin)

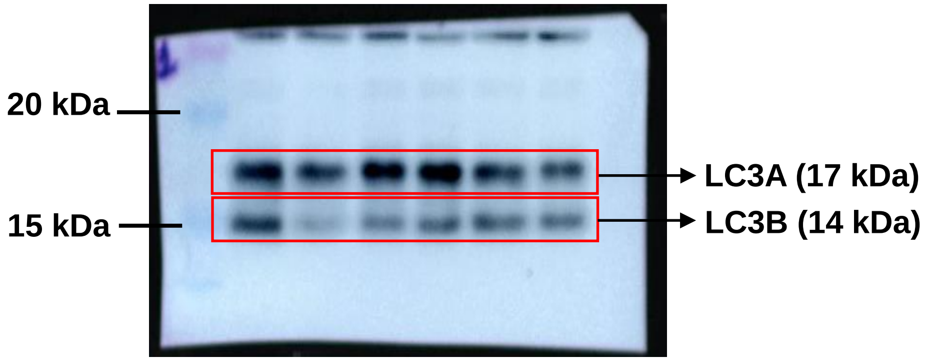

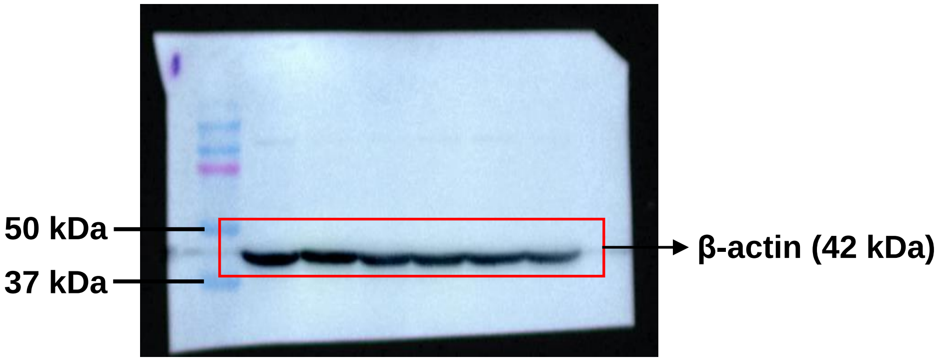

Full length blot of Fig. 7d (p-PINK1 and β-actin)

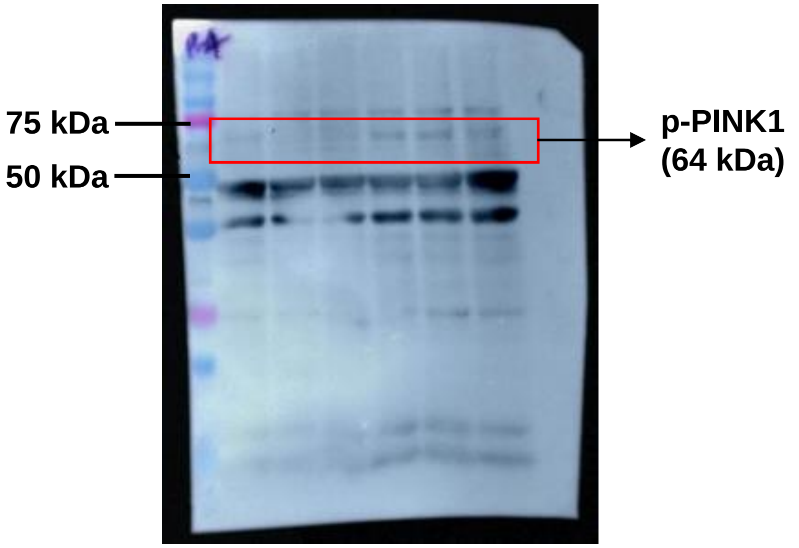

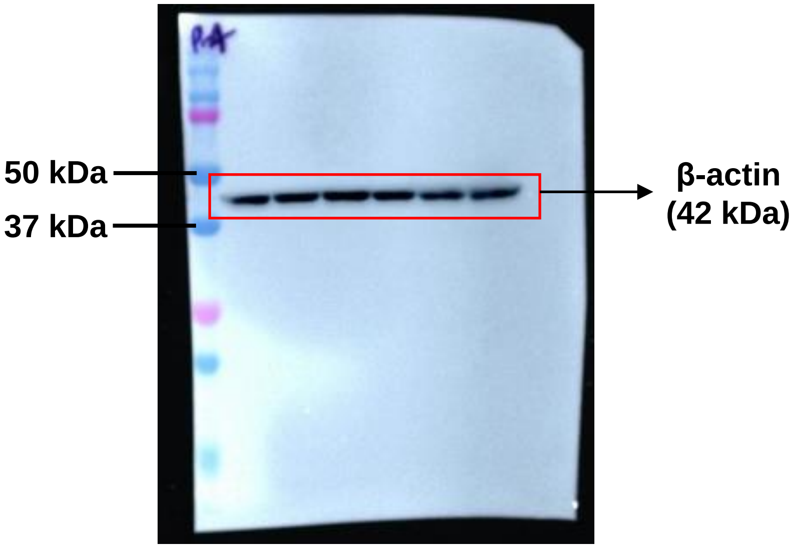

Full length blot of Fig. 7e (c-PARP1 and β-actin)

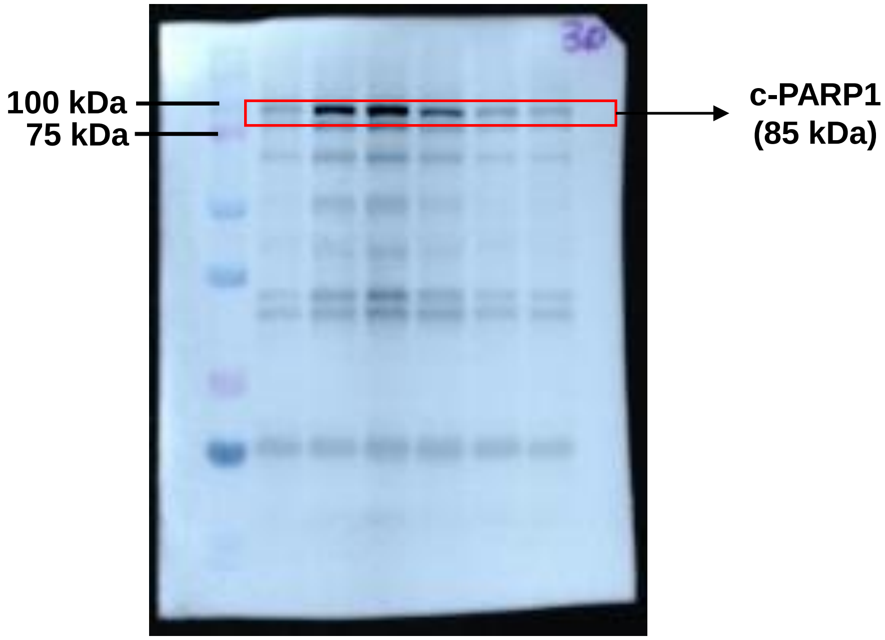

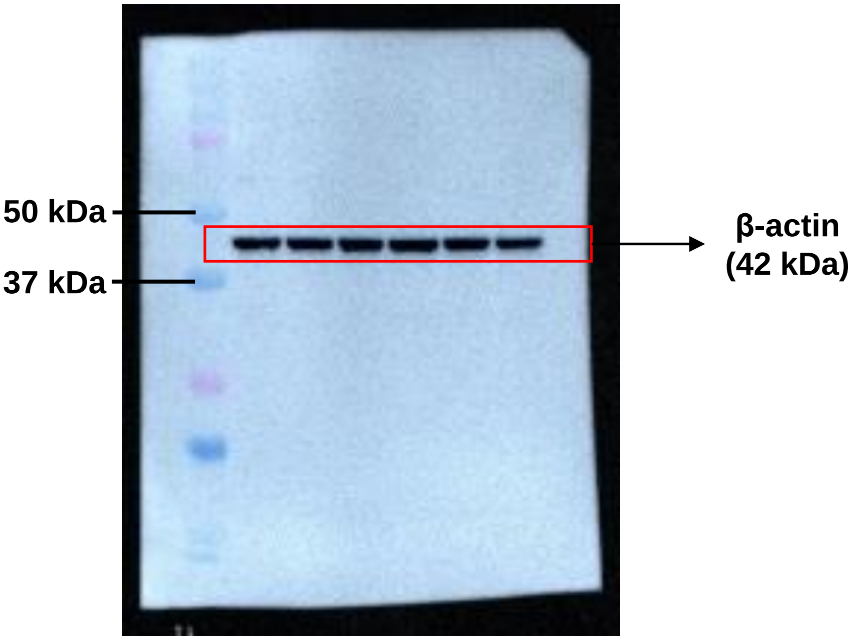

Full length blot of Fig. 8c (LXR-α and β-actin)

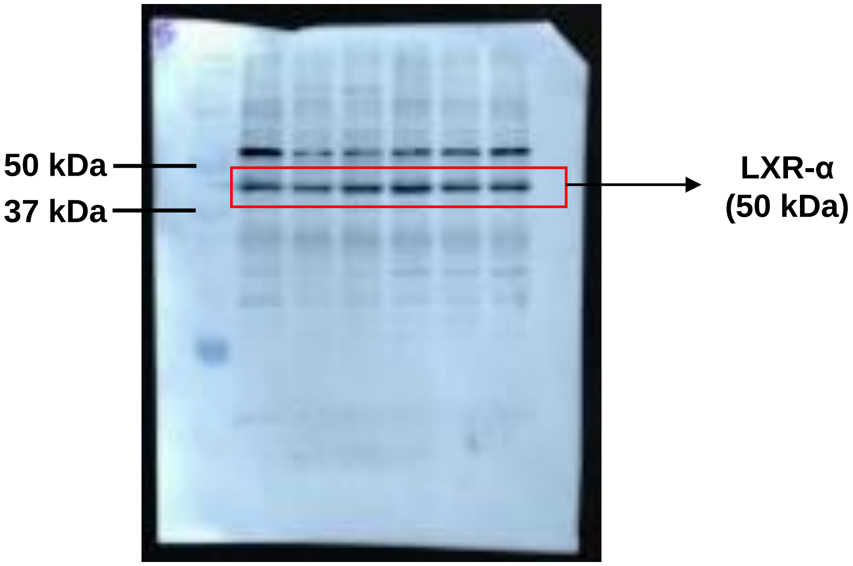

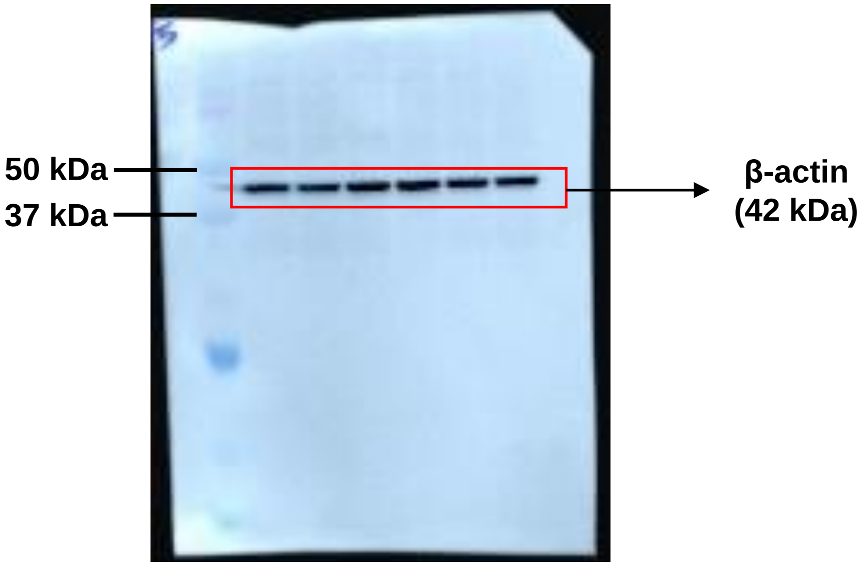

Supplement: Supplementary file 1 — Supplementary Figures. [file 41598_2024_69797_MOESM1_ESM.pdf]
